# Attenuation of melanogenesis by *Nymphaea nouchali* (Burm. f) flower extract through the regulation of cAMP/CREB/MAPKs/MITF and proteasomal degradation of tyrosinase

**DOI:** 10.1038/s41598-018-32303-7

**Published:** 2018-09-17

**Authors:** Md Badrul Alam, Arif Ahmed, Md Abdul Motin, Sunghwan Kim, Sang-Han Lee

**Affiliations:** 1 Department of Food Science and Biotechnology, Graduate School, Kyungpook National University, Daegu, 41566 Korea; 2 Food and Bio-Industry Research Institute, Inner Beauty/Anti-Ageing Center, Kyungpook National University, Daegu, 41566 Korea; 3 Department of Chemistry, Kyungpook National University, Daegu, 41566 Korea; 4 Institute of Material Chemistry, Vienna University of Technology, Vienna, Austria; 5 Green-Nano Materials Research Center, Kyungpook National University, Daegu, 41566 Korea

## Abstract

Medicinal plants have been used to treat diseases from time immemorial. We aimed to examine the efficacy of the ethyl acetate fraction of *Nymphaea nouchali* flower extract (NNFE) against melanogenesis process, and the underlying mechanisms *in vitro* and *in vivo*. Paper spray ionisation mass spectroscopy and (+) mode electrospray ionisation revealed the presence of seven flavonoids, two spermidine alkaloids, 3,4,8,9,10-pentahydroxy-dibenzo[b,d]pyran-6-one, and shoyuflavone C in NNFE. NNFE (100 µg/mL) significantly inhibited the monophenolase and diphenolase activities of mushroom tyrosinase at 94.90 ± 0.003% and 93.034 ± 0.003%, respectively. NNFE significantly suppressed cellular tyrosinase activity and melanin synthesis *in vitro* in melan-a cells and *in vivo* in HRM2 hairless mice. Furthermore, NNFE inhibited tyrosinase (TYR), tyrosinase-related protein (TYRP)-1, TYRP-2, and microphthalmia-associated transcription factor (MITF) expression, thereby blocking melanin synthesis. In particular, NNFE suppressed cAMP production with subsequent downregulation of CREB phosphorylation. Additionally, it stimulated MAP kinase phosphorylation (p38, JNK, and ERK1/2) and the proteasomal debasement pathway, leading to degradation of tyrosinase and MITF and the suppression of melanin production. Moreover, selective inhibitors of ERK1/2, JNK, and p38 attenuated NNFE inhibitory effects on melanogenesis, and MG-132 (a proteasome inhibitor) prevented the NNFE-induced decline in tyrosinase protein levels. In conclusion, these findings indicate that NNFE is a potential therapy for hyperpigmentation.

## Introduction

Melanin protects the skin from ultraviolet (UV)-induced skin damage and plays a crucial role in maintaining skin colour. However, numerous cosmetically undesirable sequelae, including freckles, chloasma, dermatitis, and geriatric skin pigmentation, result from overproduction and accumulation of melanin in the skin^1^. In addition, vitiligo, a depigmentation disorder resulting from the loss of melanocytes, is not merely a cosmetic problem but a major social problem^2^. This represents a distinct clinical challenge for both physicians and scientists to regulate melanogenesis (melanin biosynthesis) in the human epidermis. Skin pigmentation can be regulated at various stages, including melanogenic enzyme expression and regulation during or before melanogenesis, melanosome transfer to recipient keratinocytes, and melanosome degradation and turnover^3^. The expression of melanogenic enzymes is distinctly regulated by environmental factors (e.g., UV radiation [UVR]) or endogenous factors (e.g., hormones). UVR can aggravate melanin production in melanocytes either by directly affecting the melanocytes or by stimulating keratinocytes to release α-melanocyte-stimulating hormone (α-MSH), which can, in turn, upregulate tyrosinase (TYR) mRNA expression^4^. Previous studies have shown that UVR-induced increases in reactive oxygen species (ROS)/reactive nitrogen species (RNS) formation results in trigger the melanin synthesis, perhaps *via* upregulation of tyrosinase activity and increased mRNA and protein levels in melanocytes^5^. In addition, ROS/RNS contribute to melanocyte proliferation and transformation, which eventually promotes to melanogenesis^6^.

Several factors regulate melanogenesis, including melanocortin-1 receptor (MC1R), microphthalmia-associated transcription factor (MITF), adenylate cyclase/3′,5′-cyclic adenosine monophosphate (cAMP), and mitogen-activated protein (MAP) kinases. In particular, binding of α-MSH to MC1R in melanocytes trigger the stimulation of cAMP-dependent protein kinase A (PKA), which regulates the cAMP response element-binding protein (CREB), resulting in induction of MITF. MITF activation subsequently activates the melanogenesis-regulated gene coding for tyrosinase. In addition, MITF plays a critical role in melanocyte proliferation, survival, and function^7^. MAP kinases, including extracellular signal-regulated protein kinase (ERK), p38, and c-jun *N*-terminal kinase (JNK), have diverse cellular activities and important regulatory roles in melanogenesis^8,9^. Activation of ERK and p38 inhibits melanin production by sustained MITF phosphorylation leading to its degradation, as well as tyrosinase degradation^8,10^. Furthermore, the pH of melanosomes is considered a key factor in melanogenesis. Cheli *et al*.^11^ showed that cAMP activation by α-MSH or forskolin regulated vacuolar ATPase and ion transporters of the solute carrier family. This could lead to extensive changes in the ionic equilibrium of the melanocytes, resulting in alkalization of melanosomes, and thereby facilitating melanogenesis.

Numerous biological reducers and TYR inhibitor, such as kojic acid^12^, sulphite^13^, and arbutin^14^, have been developed to ameliorate hyperpigmentation disorders and pathological skin discoloration. However, whitening agents containing strong TYR inhibitors have serious adverse effects and problems, such as highly cytotoxic and unstable to oxygen and water, which limit their application. Thus, owing to their modest toxicity and favourable side effect profile, natural ingredients are now being considered in the field of cosmetic research and development of safe and efficacious skin depigmenting compounds. *Nymphaea nouchali* (Burm. f), locally known as ‘Shapla’ in Bangladesh, is an aquatic plant of the genus *Nymphaea*. *N. nouchali* grows abundantly as a mixed population in nearly all shoal natural water bodies and has been denominated as the national flower of Bangladesh. In Ayurveda, It is used as a remedy for liver disorders. The flowers, roots, and leaves, have been used for the treatment of diabetes, blood disorders, heart diseases, and dysentery, as well as a cardiotonic, diuretic, narcotic, and aphrodisiac agent^15,16^. The rhizomes and flowers have been used for treatment of kidney problems^17^. The seeds, flowers, and leaves have been shown to exhibit antioxidant, antidiabetic, antimicrobial, and haemolytic activities^18,19^. Furthermore, a novel Ca^2+^-dependent lectin, isolated from the tuber of *N. nouchali*, was found to exhibit antiproliferative properties^20^. However, the potential dermatological applications of the flowers of *N. nouchali* have not been investigated yet. To extend the efforts made to develop novel and useful cosmetic ingredients, supplements, and functional foods, the current study designed to explore the effects of *N. nouchali* flower extract (NNFE) on melanogenesis in a melanocyte cell culture system and the HRM-2 hairless mouse model. We hypothesized that NNFE functionally involve in the cAMP/p-CREB-mediated downregulation of MITF, which results in the suppression of the expression of the melanocyte-derived enzymes, TYR, TRP-1, and TRP-2. Here, we inquired whether NNFE ameliorates melanogenesis through the suppression of melanogenesis-specific enzymes via upstream events in melan-a cells and the HRM-2 hairless mouse. In addition, we investigated the anti-melanogenic activity of NNFE through the participation of MAP kinase phosphorylation as well as the NNFE-associated proteasomal abasement machinery of TYR to affirm its repression potential in melan-a cells

## Results

### Identification of the major polyphenolics in NNFE

**Figure 1:**
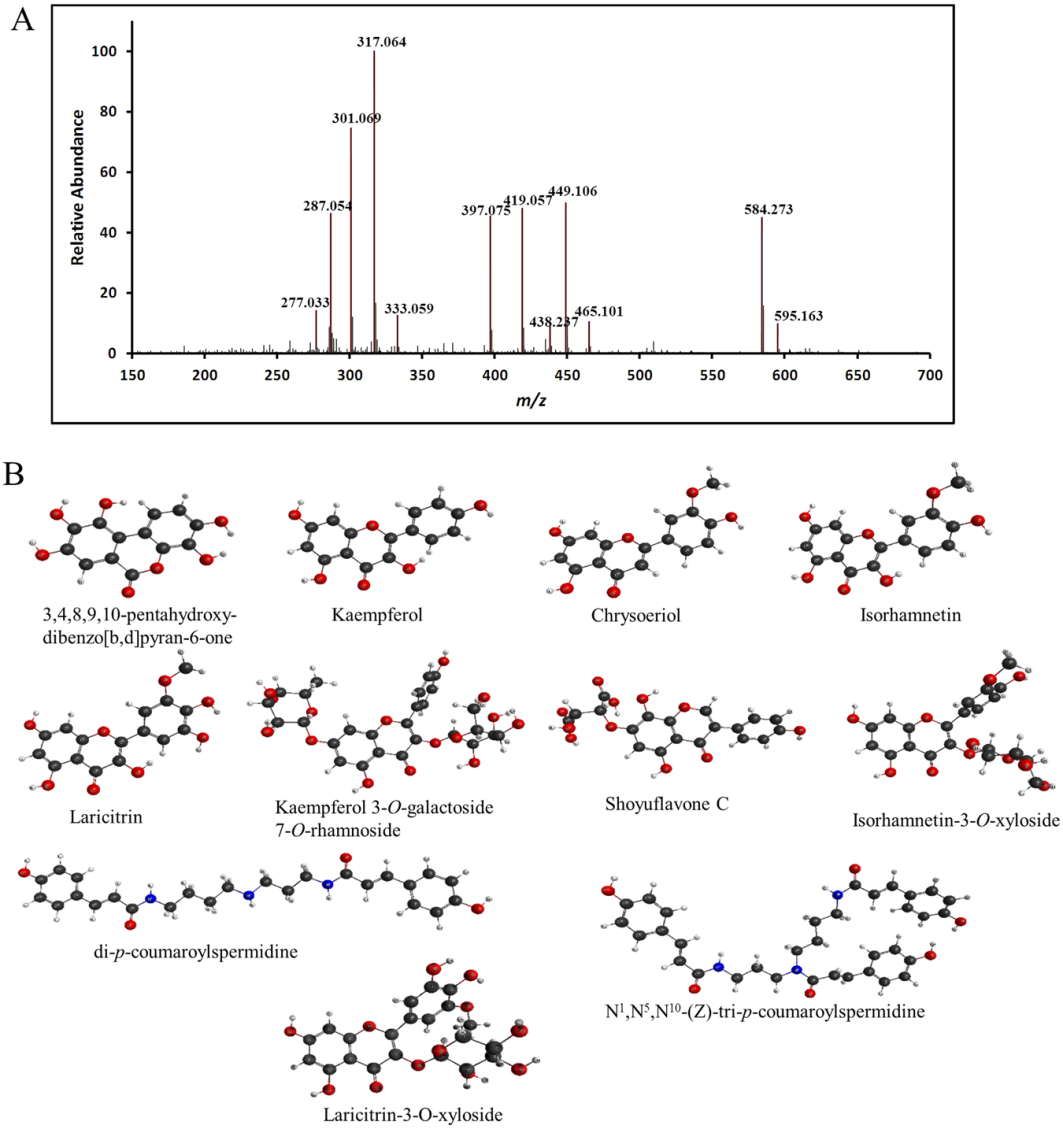
Representative paper spray ionization mass spectroscopy profile of *N. nouchali* flower extract. Paper spray ionisation mass spectrum (**A**) and structures of the identified compounds (**B**) are shown.

Identification and characterisation of the compounds isolated from NNFE were performed in two steps. In the first step, paper spray ionisation (PSI) mass spectroscopy (MS) was used to identify the major m/z peaks. The spectrum was obtained with a full-scan MS. Details are specified in the MS analysis section. The acquired spectrum is shown in Fig. 1. In the second step, the obtained m/z peaks were characterised. For characterisation, the MS/MS fragmentation pattern was determined using the electrospray ionisation (ESI) technique. MS/MS analyses for all the targeted m/z values were performed at three different normalised collision energy (NCE) values (i.e., 10, 30, and 50). High NCE values, such as 30 and 50, produce only the spectra of the product ions without precursor ions. However, low NCE values, such as 10, produce the peaks of the precursor ions, which confidently provide the origin of each product ion in a spectrum. The fragmentation patterns of the identified compounds are listed in Table 1. Compounds were tentatively identified by matching their masses and fragmentation patterns with information available in the literature. In the present study, seven flavonoids, including flavonols (kaempferol, isorhamnetin, laricitrin), flavones (chrysoeriol), and flavonol glycosides (kaempferol-3-*O*-galactoside-7-*O*-rhamnoside, laricitrin-7-*O*-xyloside, isorhamnetin-3-*O*-xyloside), were identified. Moreover, 3,4,8,9,10-pentahydroxy-dibenzo[b,d]pyran-6-one and shoyuflavone C were identified. In addition, two spermidine alkaloids (di-p-coumaroylspermidine and *N*^1^,*N*^5^,*N*^10^-(z)-tri-p-coumaroylspermidine) were identified for the first time in this genus (Fig. 1). The MS spectra generated for all the identified compounds by ESI-MS in the (+) ion mode provided the protonated molecules, (M + H)^+^. Peak 1 was identified as 3,4,8,9,10-pentahydroxy-dibenzo[b,d]pyran-6-one, *m/z* 277.035 (M + H)^+^, and yielded fragment ions at 259.02 and 231.02, corresponding to [M + H-H_2_O]^+^ and [M + H-H_2_O-CO]^+^, respectively. Peaks 2, 3, 4, and 5 were identified as kaempferol, m/z 287.0555 (M + H)^+^; chrysoeriol, m/z 301.0712 (M + H)^+^; isorhamnetin, m/z 317.0666 (M + H)^+^; and laricitrin, m/z 333.0612 (M + H)^+^, respectively. Kaempferol yielded major fragment ions at m/z 153.01 and 121.02 owing to ^1,3^A^+^ and ^0,2^B^+^ fragmentation, respectively (Supplementary Fig. S2). Mounting evidence has suggested that flavone aglycone was involved in the cleavage of two C-C bonds at positions 1/3, 0/2, and 0/4 of the C ring, which resulted in structurally informative ^1,3^A^+^ and ^1,3^B^+^ ions with corresponding m/z values of 153 and 119, respectively^21^. Furthermore, chrysoeriol, isorhamnetin, and laricitrin yielded major fragmentation ions at m/z 286.04, 301.03, and 317.02, respectively, owing to the loss of the methyl group. The MS/MS data showed peak 3 contained fragment signals at m/z 153.01 and 121.02 from ^1,3^A^+^ and ^0,2^B^+^ fragmentation, respectively, and a signal at m/z 258 (M + H-CH_3_-CO), caused by the natural loss of CO, which may be attributed to the degradation of the C ring of flavone^21^ (Supplementary Fig. S3). Furthermore, the MS/MS data for peak 4 contained fragment signals at m/z 153.01 and m/z 137 from ^1,3^A^+^ and (^0,2^A^+^-CO) fragmentation, respectively, whereas the signal at m/z 245 could be explained by the successive loss of two molecules of CO (M + H-CH_3_-2CO) and the signal at m/z 229 corresponded to the successive loss of H_2_O and two molecules of CO (M + H-CH_3_-H_2_O-2CO) (Supplementary Fig. S4). Moreover, the MS/MS data for peak 5 contained fragments signals at m/z 153.01 and m/z 137 from ^1,3^A^+^ and (^0,2^A^+^-CO) fragmentation, respectively, whereas the signal at m/z 245 could be explained by the successive loss of two molecules of CO (M + H-CH_3_-2CO) from the flavone aglycone (Supplementary Fig. S5). Peak 7 was identified as shoyuflavone C, m/z 419.0597 (M + H)^+^, and yielded a major fragmentation ion at m/z 287.05, which confirmed the presence of 8-dihydroxy genistin^22^ (Supplementary Fig. S6). It is important to note that cleavage at the glycosidic O-linkages and the concomitant H-rearrangement led to the elimination of monosaccharide residues, i.e. losses of 162 u (hexose), 146 u (deoxyhexose), 132 u (pentose), or 172 u (uronic acid), which allowed the determination of the carbohydrate sequence^23^. Peaks 9 and 10 were identified as isorhamnetin-3-*O*-xyloside, m/z 449.1090 (M + H)^+^ and laricitrin-7-*O*-xyloside, m/z 465.1039 (M + H)^+^, respectively, yielding major fragmentation ions at m/z 317.06 and 333.06, respectively, owing to the losses of xyloside (132 u) through the *O*-glycosidic cleavage. Kaempferol-3-*O*-galactoside-7-*O*-rhamnoside, m/z 595.1667 (M + H)^+^ (peak 12) yielded major fragmentation ions at m/z 449.1 and 287.05 owing to the losses of galactoside (146 u) and rhamnoside (162 u), respectively, through the cleavage of the *O*-glycosidic bonds at the 7 and 3 positions, respectively. Peaks 8 and 12 were identified as di-p-coumaroylspermidine, m/z 465.1039 (M + H)^+^ and *N*^1^,*N*^5^,*N*^10^-(z)-tri-p-coumaroylspermidine, m/z 465.1039 (M + H)^+^, respectively, and yielded major fragmentation ions at m/z 292.20 and 438.23, respectively, owing to the loss of the coumaroyl group. The MS/MS spectra of all the identified compounds are shown in the supporting information, Figs S1–S11.

**Table 1:** Peak assignments and tentative identification of the major constituents of *N. nouchali* flower extract by a combination of (+) mode PSI and ESI (MS/MS).

Compound Name	MW	Elemental formula	(M + H)^+^ m/z observed	(M + H)^+^ m/z calculated	Positive ionization MS/MS
3,4,8,9,10-pentahydroxy-dibenzo[b,d]pyran-6-one	276.20	C_18_H_8_O_7_	277.035	277.034	259.02, 231.02, 147.04
Kaempferol	286.23	C_15_H_10_O_6_	287.0555	287.055	230.98, 213.05, 153.01, 121.02
Chrysoeriol	300.26	C_16_H_12_O_6_	301.0712	301.071	286.04, 258.05, 213.05, 153.07, 121.02
Isorhamnetin	316.26	C_16_H_12_O_7_	317.0666	317.066	301.03, 245.04, 153.01, 137.02
Laricitrin	332.25	C_16_H_12_O_8_	333.0612	333.06	317.02, 301.03, 245.04, 153.01, 137.02
Unknown			397.0773		
Shoyuflavone C	418.35	C_19_H_14_O_11_	419.0597	419.053	287.05, 153.01, 107.04
di-*p*-coumaroylspermidine	437.24	C_25_H_31_N_3_O_4_	438.2402	438.239	292.2, 218.11, 204.1, 147.04, 119.04
Isorhamnetin-3-*O*-xyloside	448.26	C_21_H_20_O_11_	449.109	449.108	317.06, 287.05, 153.01, 137.02
Laricitrin-7-*O*- xyloside	464.26	C_21_H_20_O_12_	465.1039	465.103	333.06, 317.02, 301.03, 245.04, 153.01
*N*^1^,*N*^5^,*N*^10^-(z)-tri-p-coumaroylspermidine	583	C_34_H_37_N_3_O_6_	584.2769	584.268	438.23, 292.20, 218.11, 204.10, 147.04
Kaempferol-3-*O*-galactoside-7-*O*-rhamnoside	594.52	C_27_H_30_O_15_	595.1667	595.166	449.1, 287.05, 153.01, 121.02

### Mushroom tyrosinase inhibition effects by NNFE

**Figure 2:**
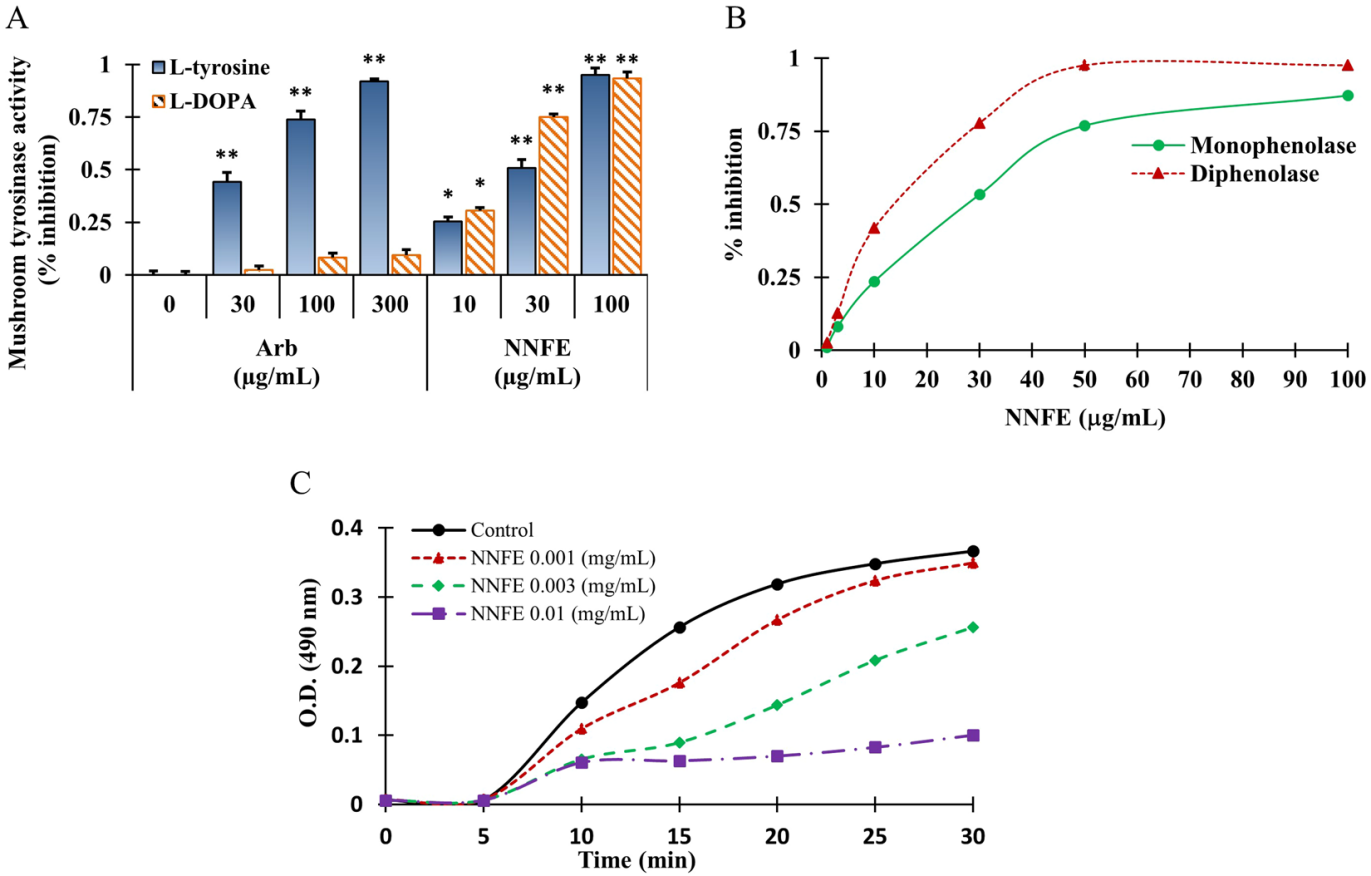
Inhibitory effects of NNFE on mushroom tyrosinase activity. (**A**) Different concentrations of NNFE or arbutin were incubated with mushroom tyrosinase. After incubation, the amount of dopachrome produced was determined spectrophotometrically at 490 nm. (**B**) Effects of NNFE on the monophenolase and diphenolase activities of tyrosinase. Enzyme activity was tested in the presence of L-tyrosine and L-DOPA, as a substrate for monophenolase and diphenolase activities, respectively. **(c)** Effects of NNFE on the monophenolase activity of tyrosinase. Enzyme activity was tested in the presence of L-tyrosine, as a substrate Results are presented as the means ± SDs of three experiments. *p < 0.05, **p < 0.01, versus the non-treated controls, Student’s *t*-test. Arb, arbutin; NNFE, ethyl acetate fraction of *N. nouchali* flower extract.

Mushroom tyrosinase is commonly exploited as a key enzyme to test melanogenesis inhibitors. Therefore, to investigate the anti-melanogenic activity of NNFE, its effects on mushroom tyrosinase were first examined by quantifying the conversion of L-tyrosine to *O*-hydroxylation of tyrosine and/or oxidation of levodopa (L-DOPA) to *O*-diquinone or both in the absence or presence of NNFE^24^. NNFE suppressed mushroom tyrosinase activity in a concentration-dependent manner, with IC_50_ values of 29.30 ± 0.11 and 18.70 ± 0.15 for the substrates, L-tyrosine and L-DOPA, respectively, whereas arbutin, a positive tyrosinase inhibitor, had an IC_50_ of 165.19 ± 0.11 with L-tyrosine (Fig. 2A, Supplementary Fig. S12). Moreover, the monophenolase and diphenolase activities of tyrosinase were inhibited by NNFE in a concentration-dependent fashion (Fig. 2B).

To calculate the kinetic parameters of tyrosinase inhibition by NNFE, we examined its effects on the monophenolase- and diphenolase-activated forms of tyrosinase (Fig. 2C, Supplementary Fig. S13). After initiating the enzymatic reaction of tyrosinase, the monophenolase activity has a marked lag period^24^. Interestingly, the monophenolase activated form of tyrosinase was markedly inhibited by NNFE in a concentration-dependent fashion. Importantly, NNFE broadened the lag phase by 25 min, compared to the control, in particular at 100 μg/mL (Fig. 2C). It is noteworthy that the reaction lag period was affected by both the concentrations of enzyme and substrate presence in the reaction medium; in addition, the presence of catalytic quantities of transition metal ions or *O*-diphenols can shorten, or even abolish the lag^25^. However, some monophenolase inhibitors, such as liquorice root, have been shown to extend the lag phase^25,26^.

### Hypopigmentation effects of NNFE in melan-a cells

**Figure 3:**
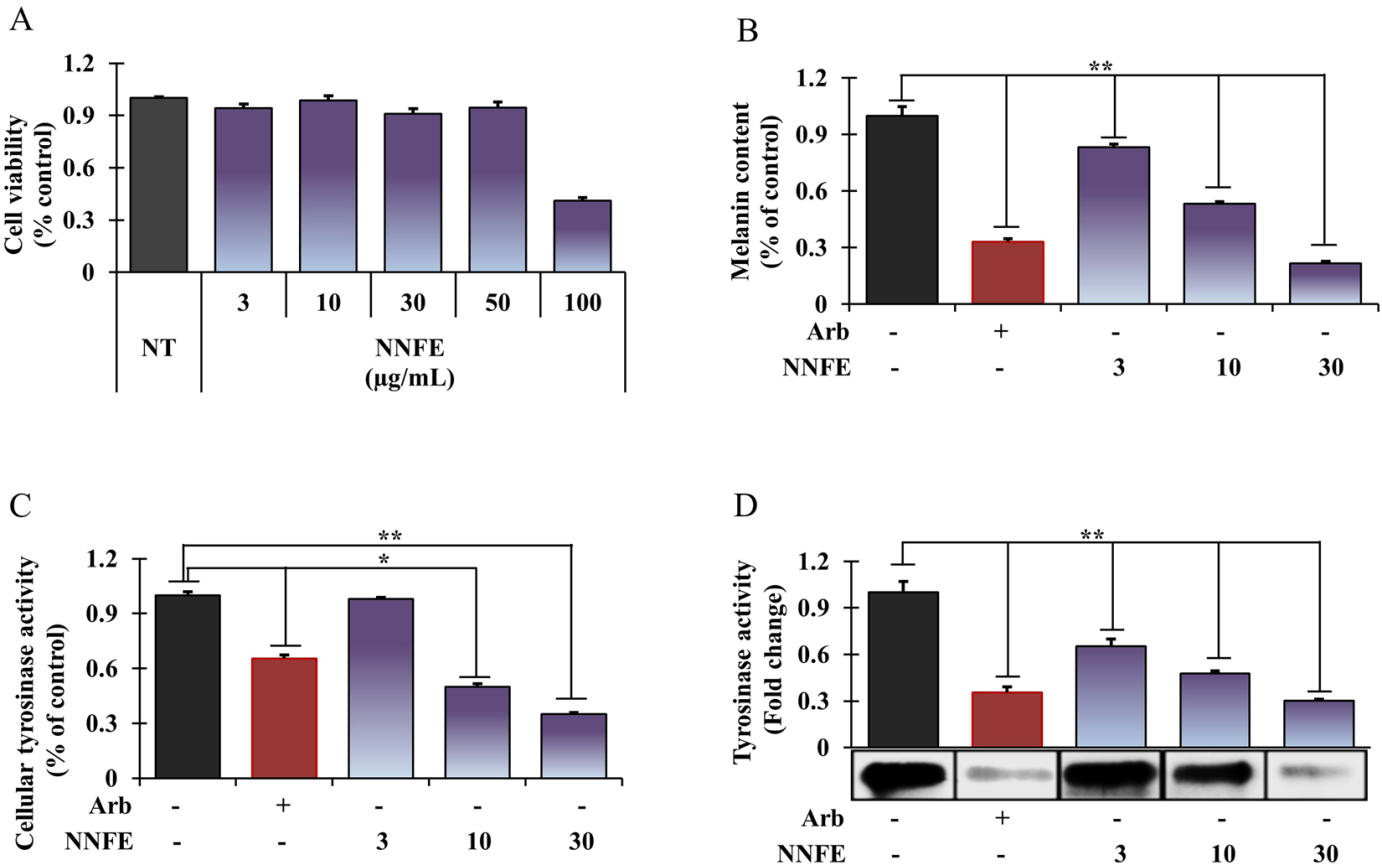
Effects of NNFE on melanogenesis in melan-a cells. Cells were cultured with NNFE (3–30 μg/mL) for 3 days. (**A**) cytotoxicity, (**B**) melanin content, (**C**) intracellular tyrosinase, and (**D**) tyrosinase activity by zymography were measured as described in the Materials and Methods. Experiments were performed in triplicate, and the results are expressed as the means ± SDs. *p < 0.05, **p < 0.01, Student’s *t*-test. NT, no treatment; Arb, arbutin; NNFE, ethyl acetate fraction of *N. nouchali* flower extract.

Melan-a cell viability and melanin content were analysed after exposure to various concentrations of NNFE (3 to 100 µg/mL). At concentrations up to 50 µg/mL, NNFE displayed no cytotoxic effects (Fig. 3A), but it significantly mitigated melanin production in a concentration-dependent manner (Fig. 3B, 3^rd^ to 5^th^ column). In addition, NNFE treatment substantially mitigated IBMX-induced melanogenesis in melan-a cells (Supplementary Fig. S14). To analyse the mechanisms underlying the repressive effects of NNFE on melanogenesis, L-DOPA zymography was carried out to assess the intracellular tyrosinase activity in the cells. As shown in Fig. 3C and D (3^rd^ to 5^th^ column), NNFE treatment strongly suppressed the cellular tyrosinase activity with an IC_50_ value of 9.85 ± 0.11 µg/mL, suggesting that NNFE can inhibit melanin synthesis in melan-a cells.

### Effects of NNFE on melanogenesis-associated proteins expression

**Figure 4:**
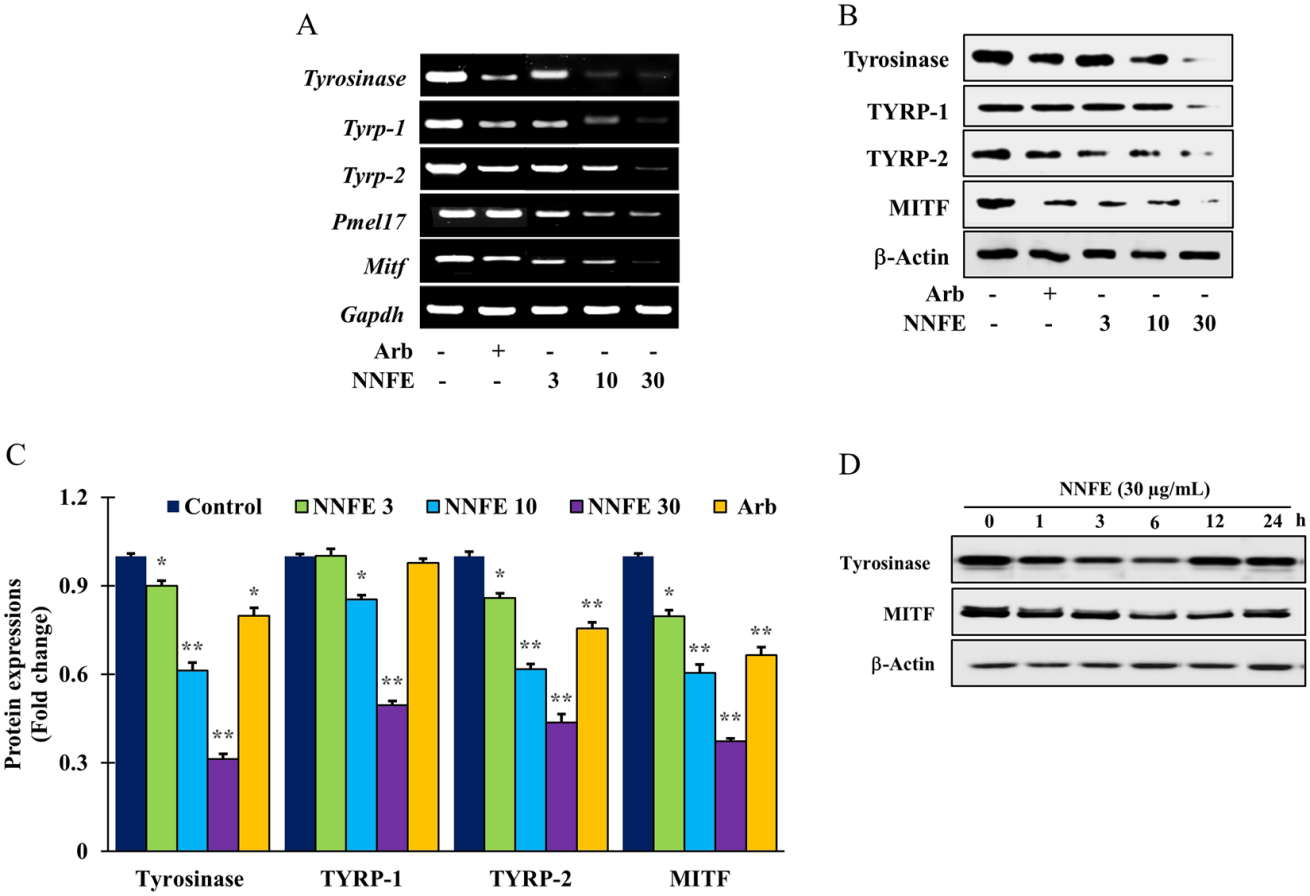
Effects of NNFE on the expression of melanogenesis-related mRNA and proteins in melan-a cells. (**A**) Cells (5 × 10^5^ cells/mL) were cultured for 24 h, and the medium was then replaced with fresh medium containing the indicated concentrations of NNFE or arbutin for 24 h. mRNA was extracted using TRIzol, and mRNA expression was determined by RT-PCR. (**B**) Cells (1 × 10^5^ cells/mL) were cultured for 24 h, and the medium was replaced with fresh medium containing the indicated concentrations of NNFE or arbutin for 3 days. Total cell lysates were extracted and assayed by western blotting using antibodies against tyrosinase, TYRP-1, TYRP-2, and MITF. Equal protein loading was confirmed using β-actin. (**C**) Statistical analysis of the band intensities of tyrosinase, TYRP-1, TYRP-2, and MITF obtained by western blot analysis. *p < 0.05, **p < 0.01, versus the non-treated controls, Student’s *t*-test. (**D**) Total cell lysates were extracted and assayed by western blotting using antibodies against tyrosinase, and MITF. Equal protein loadings were confirmed using β-actin.

To investigate whether NNFE could affect the expression of melanogenesis-concerned proteins, including TYR, tyrosinase-related protein (TYRP)-1, TYRP-2, Pmel17, and MITF, their levels were assessed in melan-a cells using reverse transcription polymerase chain reaction (RT-PCR) and western blot analysis after treatment with NNFE at different concentrations (3, 10, and 30 μg/mL) for 4 days. NNFE repressed the mRNA expression of *MITF* and its downstream genes, *tyrosinase, TYRP-1, TYRP-2, and Pmel17* (Fig. 4A, Supplementary Fig. S15). In addition, NNFE (30 μg/mL) markedly inhibited TYR, TYRP-1, TYRP-2, and MITF protein expression in a concentration-dependent manner, compared to the corresponding protein expression in the untreated controls (Fig. 4B,C, Supplementary Fig. S16). The present results suggest that NNFE suppresses the expression of MITF as well as its downstream tyrosinase-related genes and attenuates melanin biosynthesis. In addition, to determine the onset and strength of the effect of NNFE on melanogenesis-referred proteins, we first treated the cells with NNFE (30 μg/mL) and a time course of tyrosinase and MITF expression was executed. As expected, NNFE reduced the manifestation of tyrosinase and MITF from 1 to 12 h, reaching a minimum at 6 h (Fig. 4D).

The pH of the melanosomes is also considered a key factor in melanogenesis. Cheli *et al*.^11^, showed that activation of cAMP by α-MSH or forskolin regulated vacuolar ATPase and ion transporters of the solute carrier family. This could result in extensive changes in the ionic equilibrium of the melanocytes and alkalization of the melanosomes, thereby facilitating melanogenesis. Thus, to support this hypothesis, we measured the mRNA expression of various pH-regulating channels in the melanocytes. NNFE treatment considerably mitigated the mRNA expression of proton-dependent channels in melan-a cells (Supplementary Fig. S17). The data strongly suggest that reducing the pH channels contributes to the repressive effects of NNFE on melanin synthesis in melan-a cells.

### Effects of NNFE on the melanogenesis-associated signalling pathways

**Figure 5:**
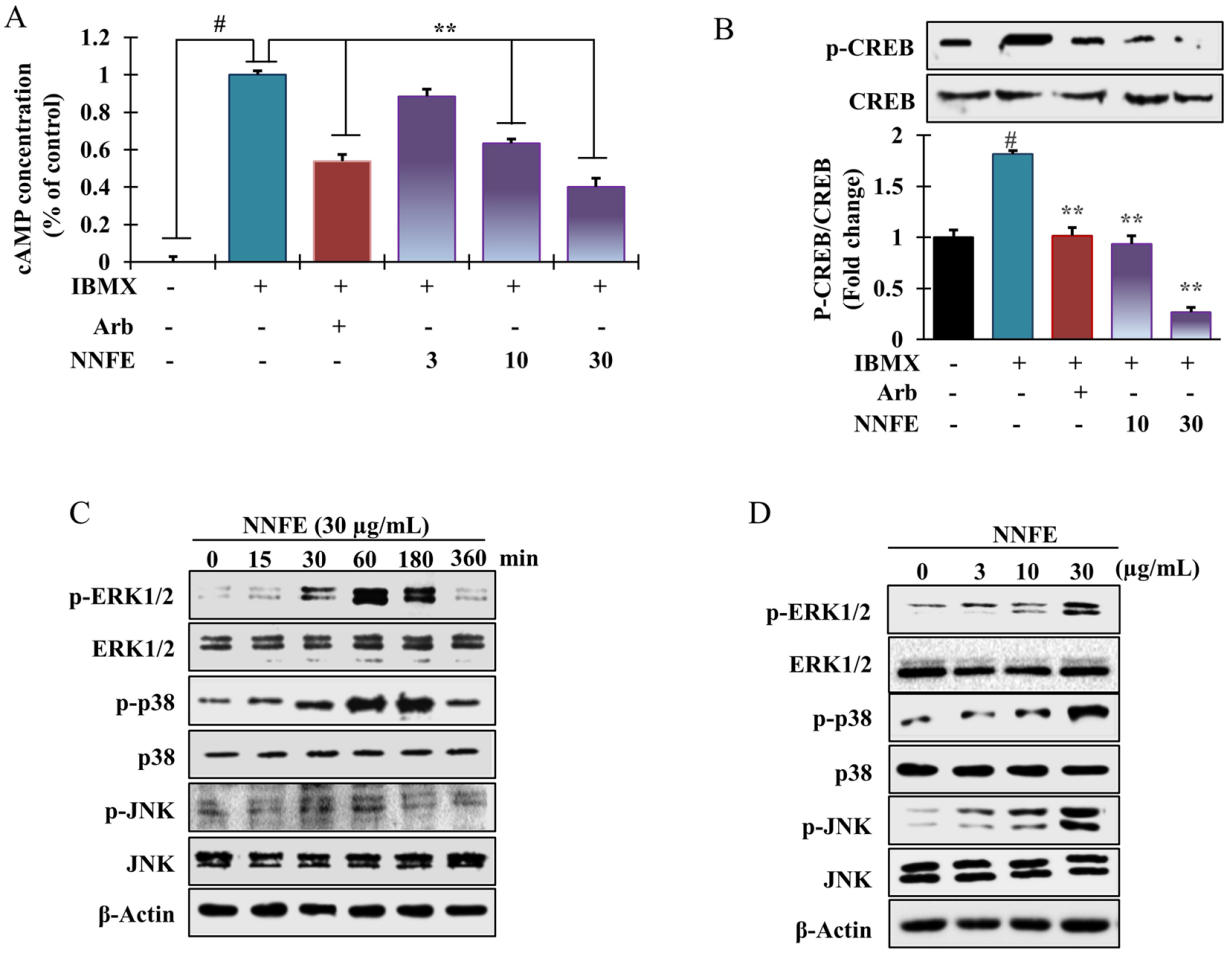
Effects of NNFE treatment on the intracellular cAMP concentration and melanogenesis-related signalling protein expression in melan-a cells. (**A**) Cells were treated with the indicated concentrations of NNFE for the indicated times. Intracellular cAMP levels were measured using a cAMP ELISA kit. Results are representative of three independent experiments. ^#^*p* < 0.05, versus the non-treated controls; **p < 0.01, versus the IBMX-treated controls, Student’s *t*-test. Western blot analysis showing the changes in p-CREB, CREB, p-ERK1/2, ERK1/2, p-p38, p38, p-JNK, and JNK expression in melan-a cells treated with **(B** and **D)** the indicated concentrations, **(C)** at the indicated times. Arb, arbutin; NNFE, ethyl acetate fraction of *N. nouchali* flower extract.

In melanogenesis, MITF is modulated by the cAMP-mediated pathway *via* CREB phosphorylation, which upregulates MITF transcription^27^. We measured the intracellular cAMP levels after NNFE treatment. NNFE decreased intracellular cAMP levels in a concentration-dependent manner (Fig. 5A), indicating that signal transduction could be inhibited by NNFE *via* reduction of intracellular cAMP levels. Furthermore, to investigate the effects of NNFE on cAMP-related signalling pathways, CREB phosphorylation was assayed by western blot. As presented in Fig. 5B, NNFE treatment markedly reduced the phosphorylated levels of CREB; however, CREB levels were unchanged. Moreover, to elucidate other mechanicses involving the anti-melanogenic effects of NNFE, melan-a cells were treated with NNFE (30 µg/mL) for the designated times, and western blotting was carried out. As presented in Fig. 5C, NNFE concentration-dependently stimulated the phosphorylation of p38, ERK1/2, and JNK from 60 min to 3 h of treatment. The above data suggest that inhibition of melanogenesis by NNFE is associated with reduced CREB phosphorylation, as well as activation of p38, ERK, and JNK signalling pathways.

**Figure 6:**
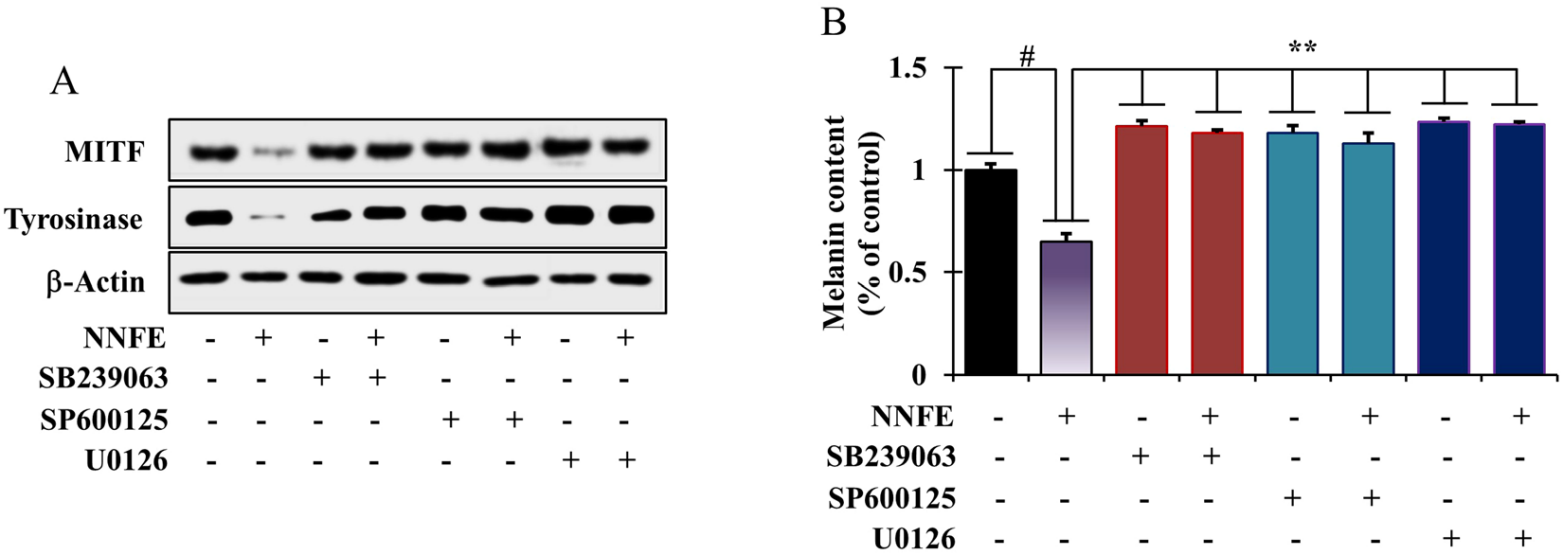
Effects of NNFE on MAP kinase signalling in melan-a cells. (**A**) Melan-a cells were co-treated with NNFE and selective inhibitors of ERK (U0126), JNK (SP600125), or p38 (SB239063). MITF and tyrosinase levels were determined by western blot analysis, and (**B**) melanin content was also determined. Measurements were made in triplicate, and results are expressed as the means ± SDs. ^#^*p* < 0.05, versus the non-treated controls; **p < 0.01, versus the IBMX-treated controls, Student’s *t*-test. NNFE, ethyl acetate fraction of *N. nouchali* flower extract.

To determine whether the increases in p38, ERK, and JNK phosphorylation were associated with the suppression of MITF expression, the cells were pre-treated with selective inhibitors of p38 (SB239063), ERK (U0126), and/or JNK (SP600125) pathways before NNFE administration. Treatment with each selective inhibitor abolished the anti-melanogenic effects of NNFE *via* inactivation of MITF and suppression of TYR manifestation (Fig. 6A). Furthermore, to ascertain the effects of NNFE-stimulated phosphorylation of p38, ERK, and JNK on melanin synthesis, melanin content was quantified in the presence of SB239063, U0126, or SP600125 in NNFE-treated cells. As anticipated, the NNFE-induced decrease in melanin content was inhibited by SB209190, U0126, and SP600125 (Fig. 6B). The present data reveal that activation of p38, ERK, and JNK pathways can mediate the anti-melanogenic effects of NNFE.

### Effects of NNFE on proteasomal degradation

**Figure 7:**
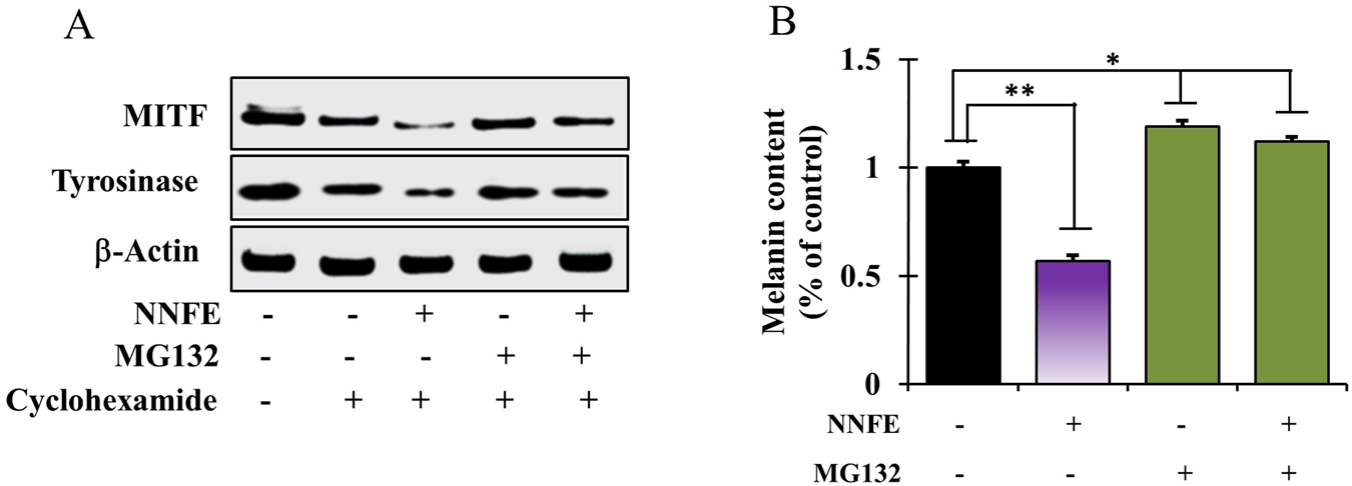
Effects of NNFE on the proteasomal degradation of tyrosinase in melan-a cells. (**A**) Cells (3 × 10^5^ cells/mL) were pre-treated with 25 μg/mL cycloheximide (a protein synthesis inhibitor) for 1 h. Separately, cells were pre-treated with 10 μM MG-132 (a proteasomal inhibitor) for 1 h. Cycloheximide and MG-132-pretreated cells were then treated with NNFE for 6 h. Whole cell lysates were subjected to western blot analysis using anti-tyrosinase antibodies. Equal protein loadings were confirmed using β-actin antibodies. (**B**) Melanin contents were determined in triplicate, and results are expressed as the means ± SDs. *p < 0.05, versus the non-treated controls, Student’s *t*-test. NNFE, ethyl acetate fraction of *N. nouchali* flower extract.

Melanogenesis is tightly regulated by the balance between the production and debasement of tyrosinase. Proteasomal degradation of TYR is believed to be involved in the turnover of tyrosinase^10^. Therefore, to scrutinize whether the NNFE-mediated downregulation of TYR is attributed to its post-translational degradation, a selective proteasome inhibitor MG-132 was used. Western blot analysis revealed that pre-treatment with MG-132 attenuated the NNFE-induced decrease in tyrosinase expression (Fig. 7A) and melanin production (Fig. 7B). Collectively, the data show that NNFE might alleviate melanogenesis by decreasing MITF and TYR levels *via* stimulation of their proteasomal degradation.

### Effects of NNFE on UVB-induced skin pigmentation in HRM-2 hairless mice

**Figure 8:**
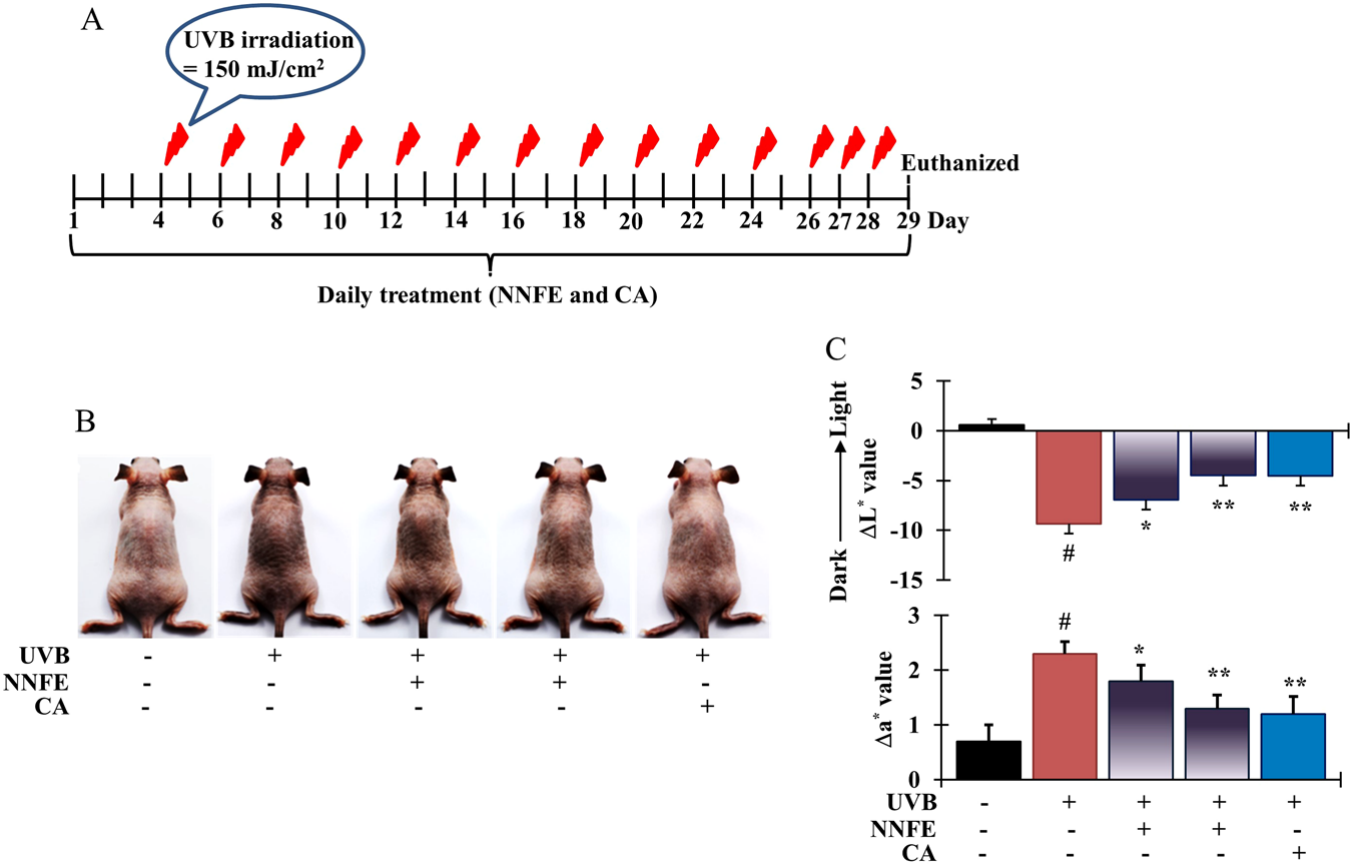
Effects of NNFE on pigmentation of mouse skin exposed to UVB. (**A**) HRM-2 melanin-possessing hairless mice were treated with the vehicle or NNFE on a designated site on the dorsal skin, according to the indicated schedules. (**B**) Representative photos show pigmentation differences in the dorsal skin among the tested animals. (**C**) The upper panel shows ΔL^*^ values calculated as the average values after the final UVB exposure minus the average baseline values before treatment. The lower panel shows Δa^*^ values, which were calculated as the average values after final UVB exposure minus the average baseline values before treatment. Results are presented as the means ± SDs. *p < 0.05, versus the non-treated controls, Student’s *t*-test. NNFE, ethyl acetate fraction of *N. nouchali* flower extract; CA, caffeic acid.

**Figure 9:**
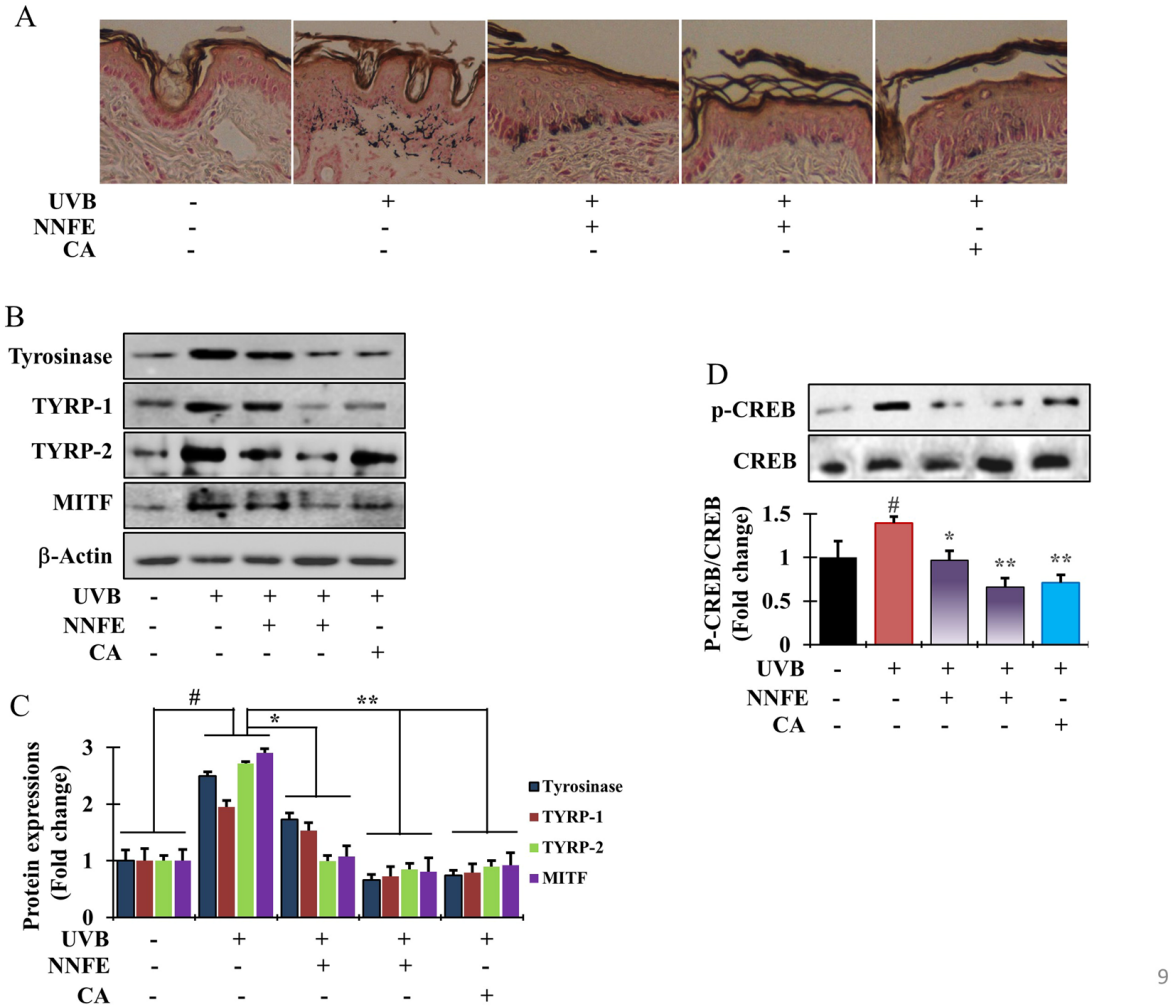
Effects of NNFE on pigmentation of mouse skin exposed to UVB. (**A**) Fontana-Masson staining of dorsal skin sections from the mice shown in (Fig. 8B) reveals differences in melanin content. (**B**) After completing UVB treatment, dorsal skin was excised, homogenised, and assayed by western blot using antibodies against tyrosinase, TYRP-1, TYRP-2, and MITF. Equal protein loading was confirmed using anti-β-actin. Arb, arbutin. (**C**) Quantification and statistical analysis of the band intensities of tyrosinase, TYRP-1, TYRP-2, and MITF obtained by western blot analysis. *p < 0.05, **p < 0.01, versus the non-treated controls, Student’s *t*-test. (**D**) Phosphorylation of CREB confirmed by western blot analysis. NNFE, ethyl acetate fraction of *N. nouchali* flower extract; CA, caffeic acid.

The inhibitory effects of NNFE on skin pigmentation *in vivo* were examined in melanin-possessing hairless mice, which were treated according to the schedule shown in Fig. 8A. Mice treated with the vehicle (control group), NNFE (50 and 100 mg/kg), or caffeic acid solution (positive control) for 3 days before UVB exposure did not show any signs of skin irritation. Repeated UVB exposure (150 mJ/cm^2^) led to visible pigmentation in the mice (Fig. 8B). The colour of the skin sites was measured using a spectrophotometer. UVB exposure led to a decrease in the L^*^ values, indicating an increase in pigmentation. The UVB-induced decrease in L^*^ values was attenuated in a dose-dependent manner in the NNFE-treated animals (Fig. 8C, top graph), indicating the potent depigmenting effects of NNFE. In addition, UVB exposure led to an increase in the a^*^ values, which are representative of sunburns. However, this UVB-induced increase in the a^*^ values was mitigated by NNFE treatment at 100 mg/kg. Fontana-Masson staining, which stains melanin^28^, confirmed the effects of NNFE. As shown in Fig. 9A, UVB-irradiated animals showed increased melanin staining. Consistent with the spectral data, the NNFE-treated animals showed reduced melanin staining, compared with that of the UVB-irradiated controls. In addition, NNFE treatment substantially decreased the expression of melanogenesis-related proteins, including TYR, TYRP-1, TYRP-2, p-CREB, and MITF, compared with that of the UVB-irradiated controls (Fig. 9B–D). These data suggest that NNFE can ameliorate UVB-induced skin pigmentation in mice, apparently through inhibition of melanogenesis.

## Discussion

Melanin biosynthesis is among the most significant factors determining human skin and hair colour. Melanogenesis is a multistage process, involving melanin synthesis, melanin transport, and melanosome release^29^. Several biological agents, such as hydroquinone, have been found that ameliorate hyperpigmentation disorders and skin discoloration. However, due to severe adverse effects such as redness, skin peeling, and vitiligo, the application of commonly used whitening agents with strong TYR inhibitor activities, such as arbutin, kojic acid, and hydroquinone, are limited^13,30^. Thus, medicinal chemists endure to explore of melanin biosynthesis inhibitors to remedy hyperpigmentation disorders, such as melasma, freckles, and age spots^14^. However, being safe and mostly exempt from adverse effects, natural constituents are now deemed worthy for developing the safe and efficacious skin depigmenting agents in the field of cosmetic research and development. In current study, NNFE was shown to hinder both the monophenolase- and diphenolase-activated form of mushroom tyrosinase. Furthermore, NNFE attenuated cellular melanin synthesis by suppressing cellular tyrosinase activity; in addition, it inhibited the expression of melanogenesis-related proteins in melan-a cells, at both the transcriptional and translational levels.

Tyrosinase acting a focal role in melanin synthesis. It catalyses the two rate-limiting steps of melanogenesis; first, the conversion of tyrosine to 3,4-dihydroxyphenylalanine (DOPA) by hydroxylation, denoted as monophenolase activity, and second, the oxidation of DOPA to form dopaquinone, which referred as diphenolase activity^31^. In the present study, both monophenolase- and diphenolase-activated forms of mushroom tyrosinase were markedly inhibited by NNFE (Fig. 2A). However, glabrene and *p*-alkoxybenzoic acid have potential in inhibiting the lag phase of monophenolase inhibition. NNFE broadened the lag phase by 25 min compared with the control, in particular at 100 μg/mL. This results led us to speculate that NNFE was bind to a site other than the active site and impeded the attachment of substrate to the enzyme through steric hindrance or through alteration of the protein conformation^32^. Furthermore, as demonstrated in Fig. 3B and C, NNFE powerfully suppressed intracellular tyrosinase activity, resulting in reduced production of melanin in melan-a cells in a concentration-dependent fashion as supported by previous scientific studies^29,33^.

Furthermore, suppression of the mRNA and protein expression of various melanogenesis-associated proteins, such as TYR, TYRP-1, and TYRP-2, is the key factor controlling melanogenesis by most TYR inhibitors^34^. We, therefore, explored the effects of NNFE on the transcription and translation of these genes in melan-a cells. Expectedly, NNFE substantially reduced the expression of MITF, which is profoundly associated with melanogenesis process as well as the MITF downstreaming enzymes, such as TYR, TYRP-1, and TYRP-2 (Fig. 4). The above results evoke that NNFE can reduce melanogenesis event by suppressing the expression of TYR, TYRP-1, and TYRP-2 *via* inactivation of MITF. Recent studies have accounted that phenolic acid such as caffeic and gallic acid effectively inhibits TYR^35,36^. The leaves extract of *Morus alba*, a well-known traditional medicine, also potently suppress the tyrosinase activity, possibly due to the abundance of polyphenolics in the extract^37^.

Although melanogenesis-associated proteins play climacteric roles in melaninogenesis, some other non-enzymatic reactions can also regulate melanogenesis process. A recent study showed that debasement of MITF can result in inhibition of the α-MSH-induced cAMP-dependent melanogenesis in melanoma cells^38^. Consequently, we hypothesised that NNFE-induced MITF downregulation is also involved in the cAMP signal transduction cascade. Expectedly, NNFE concentration-dependently decreased intracellular cAMP levels (Fig. 5A). Elevation of cAMP levels can induce the phosphorylation of CREB, which has been found to induce MITF transcription^27^. Therefore, we further explored whether NNFE could regulate downstream cAMP signalling. Interestingly, we found that, in accordance with its concentration-dependent inhibitory effects on cAMP, NNFE also downregulated CREB phosphorylation (Fig. 5B). A recent study revealed that *Arthrophytum scoparium*, a small shrub, extract exerted TYR and TYR-regulated gene repression, preponderantly owing to the downregulation of MITF and Mc1R^39^. Mounting evidence suggests that the MAPKs (ERK, p38, and JNK) exert significant governing functions in melanogenesis^40^. Scientific reports have shown that melanogenesis inhibitors induce phosphorylation of ERK, JNK, and p38, which results in MITF phosphorylation at serine 73 and concomitant ubiquitin-dependent proteasomal degradation^41^. Therefore, we examined the phosphorylation of MAP kinases by western blot to confirm the underlying mechanism of the anti-melanogenic action of NNFE. At nontoxic concentrations, NNFE was able to stimulate the phosphorylation of ERK, JNK, and p38 both time- and concentration-dependently (Fig. 5B and C), whereas co-treatment with an ERK1/2, JNK, or p38 inhibitor dramatically abolished NNFE-induced MITF inhibition (Fig. 6).

Selective clearance of proteins plays an important role in the regulation of homeostatic conditions in eukaryotic cells. Proteolysis by proteasomes and lysosomes is viewed as the foremost pathway of protein degradation^42^. After the post-Golgi stage, linoleic acid induces the proteasomal degradation of tyrosinase *via* post-translation alteration in the endoplasmic reticulum in melanoma cells^43^. Alternatively, endosomal/lysosomal degradation of tyrosinase was accounted in inulavosin-treated melanoma cells^44^. Indeed, debasement of tyrosinase can be hastened by anti-melanogenic compounds *via* one of the two mechanisms. In current study, NNFE-induced stifling of tyrosinase was hindered by MG-132 in melan-a cells, which indicated that NNFE-stimulated tyrosinase downregulation can be attributed to the endogenous tyrosinase debasement by proteasome (Fig. 7).

The HRM-2 melanin-possessing hairless mouse model was used as an *in vivo* model. Numerous compounds showing anti-melanogenic effects *in vitro* were not efficacious *in vivo*, possibly because of an inability to penetrate the *stratum corneum* barrier^45^. In this study, NNFE showed significant efficacy in modulating UVB-induced melanogenesis *in vivo* (Fig. 8) *via* decreased CREB phosphorylation with subsequent downregulation of the expression of MITF and TYR, TYRP-1, and TYRP-2.

Qualitative MS/MS analysis of NNFE revealed the presence of kaempferol; isorhamnetin; laricitrin; chrysoeriol; kaempferol-3-*O*-galactoside-7-*O*-rhamnoside; laricitrin-7-*O*-xyloside; isorhamnetin-3-*O*-xyloside; 3,4,8,9,10-pentahydroxy-dibenzo[b,d]pyran-6-one; shoyuflavone C; di-p-coumaroylspermidine; and *N*^1^,*N*^5^,*N*^10^-(z)-tri-p-coumaroylspermidine. A previous study suggested that flavonoids, such as kaempferol, isorhamnetin, and 8-hydroxygenistein (an isoflavone), significantly inhibit both the monophenolase and diphenolase activities of mushroom tyrosinase, in addition to their potent anti-melanogenic activities^43,44,46^. All of the identified compounds have been shown to possess anti-melanogenic activities *via* modulation of MAP kinase pathways. Hence, NNFE is a cocktail of many good therapeutics, which can display an efficient combinatorial effect in patients.

## Conclusions

**Figure 10:**
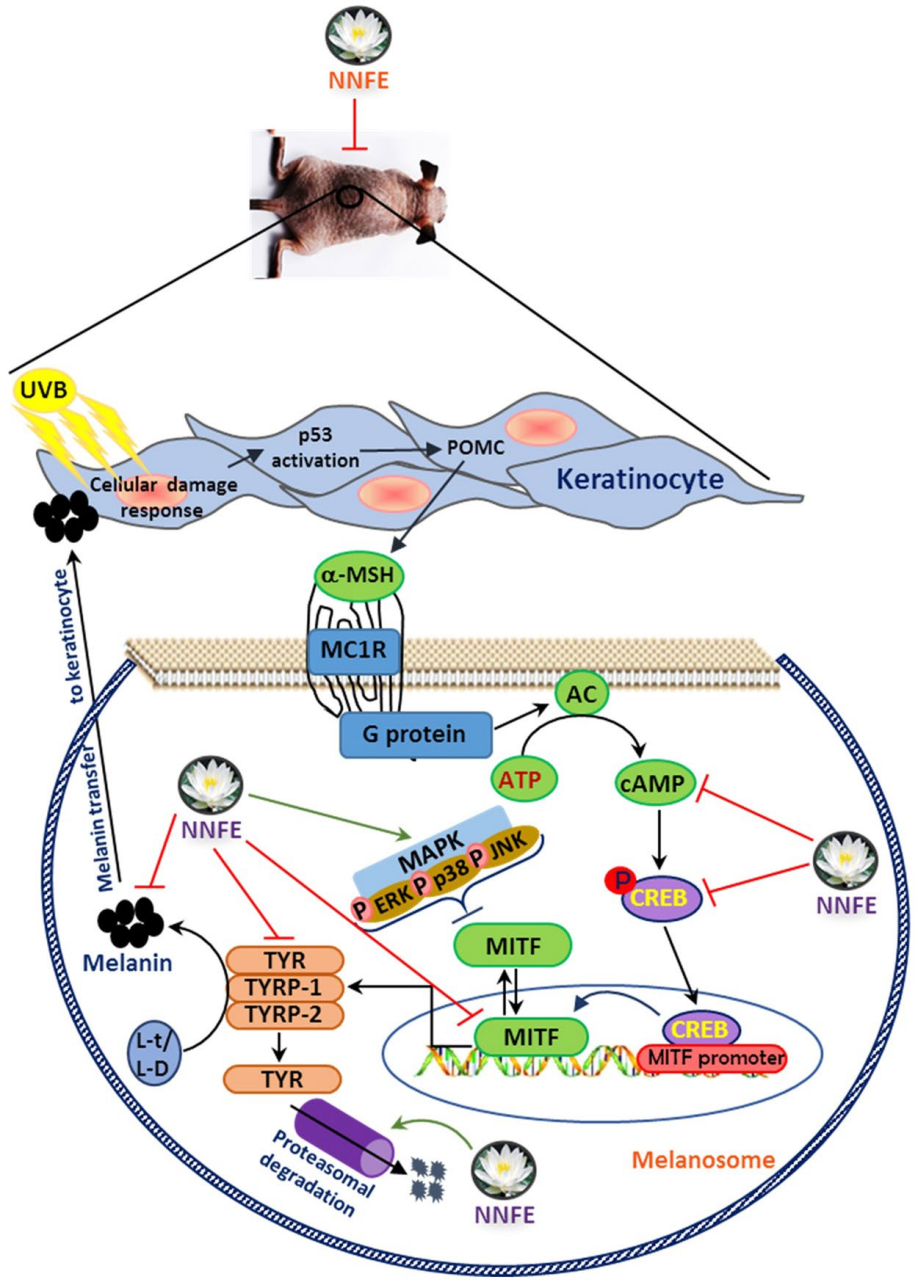
A proposed molecular mechanism of NNFE against melanogenesis process.

The current study reported the repressive effects of NNFE on melanin biosynthesis in melan-a cells and the HRM-2 hairless mouse model. The significance of this study can be summarised as follows (Fig. 10): (a) NNFE potently reduced melanogenesis and inhibited mushroom tyrosinase activity. (b) The mechanisms underlying the inhibitory effects of NNFE on melanin production involved interference with the transcription factors and common signalling pathways implicated in melanin synthesis. In particular, NNFE reduced melanin content *in vitro* and *in vivo* by downregulating MITF expression *via* suppression of cAMP, with a subsequent decrease in CREB phosphorylation and induction the phosphorylation of p38, JNK, and ERK1/2 as well as decreasing TYR, TYRP-1, and TYRP-2 levels. (c) Moreover, current discoveries evoke that the detected inhibitory effects of NNFE on melanogenesis are attributable to the endogenous tyrosinase debasement by proteasome in melan-a cells. (d) Furthermore, our data suggested that the observed suppression of melanin by NNFE might result from the presence of the bunch of polyphenolic compounds that allow for the synergistic effect. Judging from our findings and given that NNFE exhibited no cytotoxicity, we suggest that NNFE can be used as a potent natural depigmenting agent.

## Materials and Methods

### Plant materials and extraction

Fresh flowers of *N. nouchali* were collected from the lake Dakatia, in Khulna district, Bangladesh in August 2015. A senior scientist from the National Herbarium of Bangladesh was taxonomically identified the plant materials. The voucher specimen was preserved as No. 35453 and deposited in our laboratory. A 400 g of dried ground powdered flowers were subjected to extraction with methanol (3 × 1.0 L, 3 h each), filtered, evaporated under reduced pressure to obtain methanolic extract (125.36 g) of *N. nouchali* (NNFM). The obtained residue was suspended in water (500 mL) and successively partitioned with hexane, chloroform, and ethyl acetate (each 500 mL × 3) to obtain soluble fraction of hexane (NNFH, 7.25 g), chloroform (NNFC, 10.65 g), ethyl acetate (NNFE 52.32 g) and aqueous (NNFW, 37.25 g).

### Paper spray ionisation (PSI)

A Q-Exactive™ Quadrupole-Orbitrap™ mass spectrometer (Thermo Fisher Scientific Inc., Rockford, IL, USA) was used to perform PSI experiments. Methanol was purchased from Sigma-Aldrich (St. Louis, MO, USA) and used without further purification. NNFE was dissolved in methanol at a concentration of 50 μg/mL to prepare a stock solution. Then, the stock solution was diluted to a concentration of 10 μg/mL . Two microliters of the prepared solution was loaded onto a paper tip to perform the PSI experiment. (+) Mode PSI was used over a range of m/z 100–2000. Normal chromatographic paper was used in the PSI experiment. A disposable glass Pasteur pipette (Volac, Poulten & Graf Ltd, UK) was used to load the extracted sample directly onto the centre of the chromatographic paper tip. To make a sharp tip, the chromatographic paper was cut at dimensions of 6-mm base and 14-mm height. A syringe pump (Fusion 100 T, Chemyx, Stafford, TX, USA) was used to load the methanol solvent onto the sample-loaded paper. The flow rate was 40 μL/min, and the voltage directly applied on the paper for ionisation was 3–7 kV. Other parameters for PSI were as follows: spray voltage, 4.5 Kv; capillary temperature, 300 °C; mass resolution 140,000 (FWHM); maximum injection (IT) time, 150 ms; and automatic gain control (AGC), 1 E^6^.

### Electrospray ionization (ESI)

ESI was used to perform MS/MS experiments using the same spectrometer. Concentrated NNFE stock solution (50 µg/mL) was sprayed onto the ESI source at a flow rate of 40 μL/min using a 5-mL syringe (Hamilton, Reno, NV, USA). To determine the key chemical diagnostic product ions over the full range of the product ion spectrum, three different stepped NCE values (10, 30, and 50) were used. The mass spectrometer was operated in the (+) mode. Other parameters for MS/MS experiments were as follows: sheath and auxiliary gas flow rate, 10 and 0 (arbitrary units), respectively; capillary temperature, 300 °C; and S-lens, 50.

### Determination of mushroom tyrosinase activity

The assay was performed as described previously with slight modification^33^. First, a 100 mM phosphate buffer (pH 6.5) with or without sample, was added to a 96-well microplate (SPL, Pocheon, Korea) and consequently added with 200 units/mL of mushroom tyrosinase, and L-tyrosine (0.01 M), followed by incubation at 37 °C for 5 min. Then, the amount of dopachrome production was determined spectrophotometrically at 490 nm.

### Cell culture and cell viability assay

Melan-a cells (obtained from Dorothy C. Bennett, St George’s, University of London, London, UK) were maintained at 37 °C under 5% CO_2_ in Roswell Park Memorial Institute (RPMI) 1640 medium supplemented with 10% foetal bovine serum (FBS, Hyclone, Utah, UT, USA), streptomycin–penicillin (100 µg/mL each), and 200 nM 12-O-tetradecanoylphorbol-13-acetate (TPA, a potent tumour promoter). MTT (3-(4,5-dimethyl-2-thiazolyl)-2,5-diphenyl-2H-tetrazolium bromide) assay was carried out to confirm the the cytotoxicity of NNFE^29^.

### Measurement of melanin content

Cells were seeded at a density of 1 × 10^5^ cells/mL into a 24-well culture plate (BD Falcon, Bedford, MA, USA) and left to grow overnight. The old media was substituted with fresh media and treated with various concentrations of NNFE. After incubation for 3 d, cells were rewashed with PBS, lysed using 1 N NaOH. The absorbance of the sample at 405 nm was measured with a microplate reader (VICTOR3, Perkin Elmer)^9^. Inhibition of melanin biosynthesis (%) was estimated as follows:

1 $${\rm{Inhibition}}\,{\rm{of}}\,{\rm{melanin}}\,{\rm{production}}\,( \% )=({\rm{A}}-{\rm{B}})/{\rm{A}}\times {\rm{100}}$$

where A and B is the absorbance of cells lysate treated with and without of NNFE or arbutin (positive control), respectively.

### Determination of tyrosinase activity by zymography

Tyrosinase zymography was executed as described previously^9^. Cells (1 × 10^5^ cells/mL) were treated with or without NNFE for 3 d. Next, cells were rinsed with PBS twice and collect the cell lysate using RIPA buffer affixed with protease and phosphatase inhibitors cocktail (Abcam, Cambridge). An aliquot of the lysate was used to ascertain the protein content with a BCA protein assay kit (Pierce Biotechnology, Rockford, IL). The proteins (50 µg) were mixed with sampling buffer (no β-mercaptoethanol or heating) and separated using 10% SDS-polyacrylamide gel electrophoresis. Gel containing tyrosinase activity was successively reacted for 30 min in 100 mM sodium phosphate buffer (pH 6.8) with mild shaking. Finally, stain the gel with L-DOPA solution for 1 h.

### Measurement of cAMP concentration

A cAMP direct immunoassay kit (Biovision, San Francisco, CA, USA) was used to measure cellular cAMP concentrations. Cells were cultured in 6-well plates and incubated at 37 °C overnight. Various concentrations of NNFE were added to the cells, which were then lysed and harvested using 1 N HCl, according to the manufacturer’s instructions. Termination of the reaction and cAMP measurements were performed using a microplate reader, according to the manufacturer’s instructions.

### Experimental animals and UVB irradiation

Six-week-old male hairless mice (HRM-2) were obtained from Central Lab Animals, Inc. (Seoul, Korea) and were housed in an air-conditioned animal room at a temperature of 23 ± 1 °C, humidity of 55 ± 5%, and 12 h/12 h light/dark cycle with *ad libitum* access to water and standard laboratory diet. The animals were allowed to acclimatise for 1 week. They were randomly divided into five groups (five mice each). All animal experiments were conducted in accordance with the guidelines for animal experiments issued by Kyungpook National University and approved by the Institutional Animal Care and Use Committee of Kyungpook National University (KNU-2017-0025). NNFE (2.5%) was dissolved in a solution of propylene glycol and ethanol (3:7). Solutions of the positive control and/or NNFE (200 µL) were topically applied to a 3 cm × 3 cm designated area on the dorsal skin of the hairless mice once daily for the entire study period. In particular, before UVB (302 nm) exposure, mice were pre-treated with NNFE or control solution for 3 days. From day 4 to day 28, 30 min after NNFE or control solution treatment, mice were exposed to UVB (150 mJ/cm2) radiation for 10 min every other day in a UVB exposure chamber. On day 29, mice were euthanised, and the dorsal skin was removed and immediately frozen in liquid nitrogen for western blot analysis. For staining purposes, 4% paraformaldehyde solution was used to fix the excised skin. On day 29 (one day after the last NNFE dose and UVB exposure), the colour of the treated skin sites was measured using a CR-10 spectrophotometer (Konica Minolta Sensing, Inc., Sakai, Osaka, Japan). The colours were described by L^*^ (higher and lower values mean whiter and blacker colour, respectively), according to the Commission International de l′Eclairage colour system. Since no difference in skin colour was observed between the non-treated mice and control mice treated with the vehicle containing propylene glycol and ethanol, mice treated with the sham solution were considered the control group.

### Fontana-Masson staining

Fontana-Masson staining was performed to assess melanin formation in the skin of hairless mice. Fresh skin samples were fixed in 4% paraformaldehyde overnight at 27 ± 1 °C and stained using a Fontana-Masson staining kit (American Mastertech, Inc., Lodi, CA, USA). Briefly, sliced skin samples were stained with an ammoniacal silver solution for 60 min at 60 °C. The samples were incubated in 0.1% gold chloride followed by incubation in 5% sodium thiosulphate. Melanin spots were visualised using an AE-31 light microscope (Motic, Hong Kong).

### Reverse transcription polymerase chain reaction (RT-PCR)

Total RNA was purified using TRIzol (Invitrogen Co., Carlsbad, CA, USA) and aliquot (2 µg) of total RNA was reversed transcribed using RT-& GO Mastermix (MP Biomedicals, Seoul, Korea) according to the manufacturer’s instructions. A PCR Thermal Cycler Dice TP600 (TAKARA Bio Inc., Otsu, Japan) was used to carried out RT-PCR. The primer sequences are listed in Table S1. PCR products were transferred on 2% agarose gels in Tris-Acetate-EDTA (TAE) buffer at 100 V for 30 min and stained by ethidium bromide (Bio-Rad Laboratories, Hercules, CA, USA) staining. The bands were analysed using the Image Lab™ Software, version 5.2.1 (Bio-Rad laboratories, CA, USA).

### Preparation of cell lysates and western blotting

Cell lysates were mixed with SDS buffer (3 M, Maplewood, Minnesota, USA), and denatured at 100 °C for 5 min using a standard protocol. Adequate amount of proteins (30 µg) were separated using 10% SDS-polyacrylamide gel electrophoresis and blotted onto nitrocellulose membranes (Whatman, Dassel, Germany). The membranes were blocked with 5% non-fat skim milk in TBS-T buffer, followed by incubation with primary antibodies in 5% skim milk. Primary antibodies, such as anti-MITF, anti-CREB, anti-tyrosinase, anti-TRP-2, anti-p-CREB, anti-TRP-1, and anti-β-actin were purchased from Bioworld Technology (St. Louis Park, MN, USA). Anti-mouse IgG-horseradish peroxidase (HRP) and anti-goat IgG-HRP from Santa Cruz Biotechnology was used as secondary antibody. An ECL solution system (Perkin Elmer) was used to detect the antigen-antibody reaction.

### Statistical analysis

Data were analysed using one-way analysis of variance (ANOVA), and demonstrated as the means ± standard deviations (SDs). Statistical analyses were achieved using SPSS for Windows Ver. 10.07 (SPSS, Chicago, IL, USA), and significance was set at *p* < 0.01 or *p* < 0.05, as indicated.

## Electronic supplementary material

Supplementary information

## Data Availability

The authors declare that all data supporting the findings of this study are available in the article and can be provided by the corresponding author upon reasonable request.
