# Supplementary material for: Attenuation of melanogenesis by Nymphaea nouchali (Burm. f) flower extract through the regulation of cAMP/CREB/MAPKs/MITF and proteasomal degradation of tyrosinase
Source: Sci Rep. 2018 Sep 17;8:13928. doi: 10.1038/s41598-018-32303-7 (PMC6141596; doi:10.1038/s41598-018-32303-7)
Supplement: Supplementary file 1 — Supplementary information [file 41598_2018_32303_MOESM1_ESM.pdf]

SUPPLEMENTARY DATA SHEETS

Attenuation of melanogenesis by *Nymphaea nouchali* (Burm. f) flower extract through the regulation of cAMP/CREB/MAPKs/MITF and proteasomal degradation of tyrosinase

Md Badrul Alam[1,2#], Arif Ahmed[3#], Md Abdul Motin[4], Sunghwan Kim[3,5*], Sang-Han Lee[1,2*]

[1]Department of Food Science and Biotechnology, Graduate School, Kyungpook National University, Daegu 41566, Korea

[2]Food and Bio-Industry Research Institute, Inner Beauty/Anti-Ageing Center, Kyungpook National University, Daegu 41566, Korea

[3]Department of Chemistry, Kyungpook National University, Daegu 41566, Korea

[4]Institute of Material Chemistry, Vienna University of Technology, Vienna, Austria

[5]Green-Nano Materials Research Center, Kyungpook National University, Daegu 41566, Korea

[#]These authors contributed equally to this work.

[*]Correspondence to:

Dr. Sunghwan Kim; Tel.: +82-53-950-5333; E-mail: sunghwank@knu.ac.kr

Dr. Sang-Han Lee; Tel.: +82-53-950-7754; E-mail: sang@knu.ac.kr

Table S1: List of the primer sets used in this study

Gene name		***Sequences***
Tyrosinase	*forward*	*TATGCGATGGAACACCTGAG*
	reverse	*ATAGGTGCATTGGCTTCTGG*
TYRP1	*forward*	*TCACTGATGCGGTCTTTGAC*
	reverse	*TTCGGGAGCTCCTCATAGTC*
TYRP2	*forward*	*CCCGACTGTAATCGGAAGAA*
	reverse	*TCCAGCCACAACAGATGGTA*
MITF	*forward*	*ACCCGTCTCTGGAAACTTGA*
	reverse	*TCCACAGAGGCCTTGAGAAT*
Pmel 17	*forward*	*GGCACACACACAATGGAAGT*
	reverse	*GCTTCTGCAGTTGGCATGTA*
SLC24A5	*forward*	*GGGCTCTATGTTCTGCTGCT*
	reverse	*TCTCCTGCAGTCTGGTGTTG*
SLC45A2	*forward*	*CTAACCCAAGGCAGAAGCTG*
	reverse	*CGTATTCATGCATCCCACTG*
OCA2	*forward*	*GACATGCGCCTAGAGAACAA*
	reverse	*AGCAACCTCTTTACCCAGCA*
VDAC	*forward*	*TGGGAAATTAAAGGCCTCCT*
	reverse	*GCTGTCCATGCCAGGTTTAT*
TRPM1	*forward*	*AGACCATGTCCAACCCTCTG*
	reverse	*CGCAGTATTTGTGTGCGAAG*
Gapdh	*Forward*	*TTGTGATGGGTGTGAACCAC*
	reverse	*ACACATTGGGGGTAGGAACA*

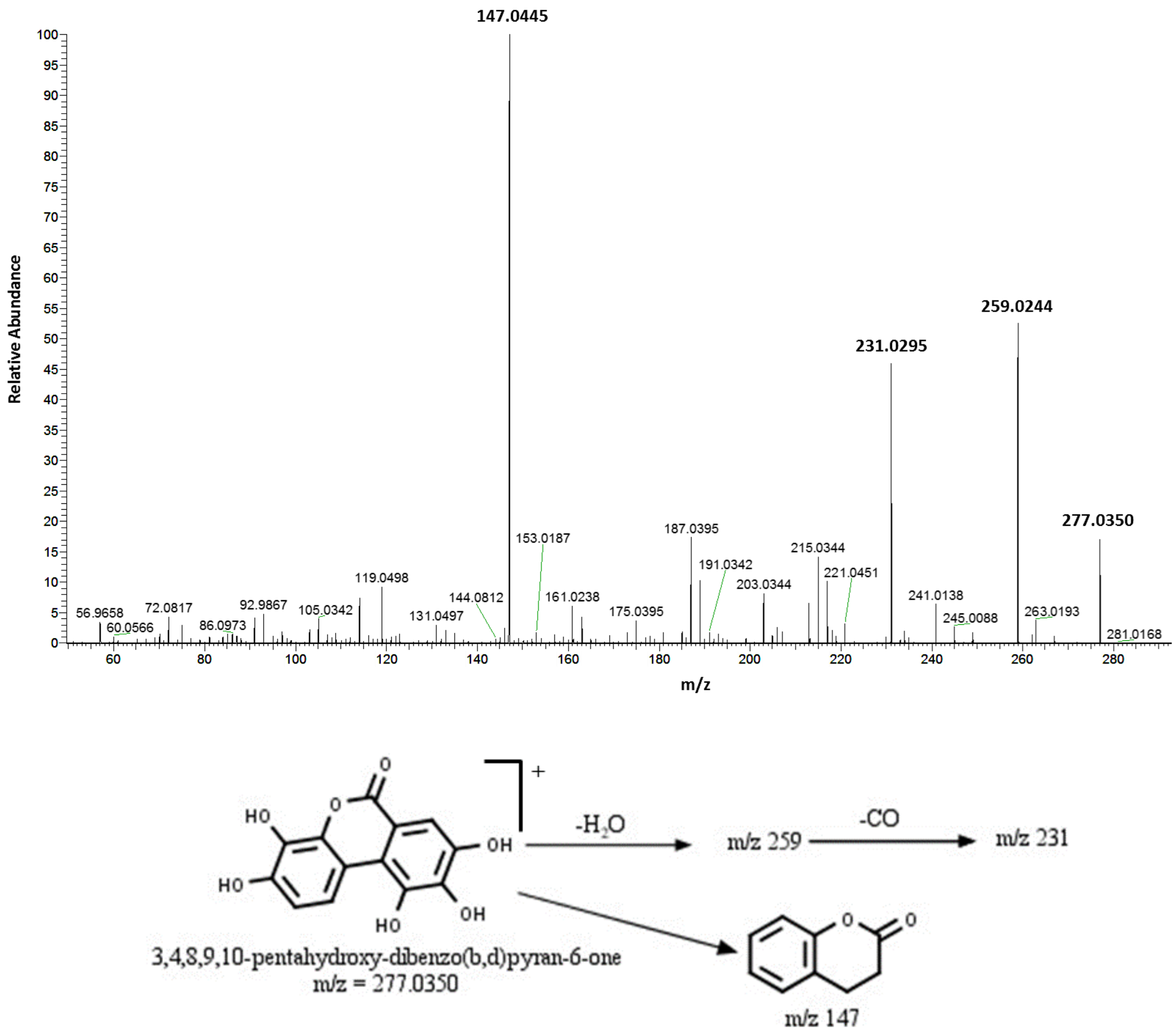

Figure S1: Chemical Structure of 3,4,8,9,10-pentahydroxy-dibenzo(b,d)pyran-6-one and possible mass fragmentation

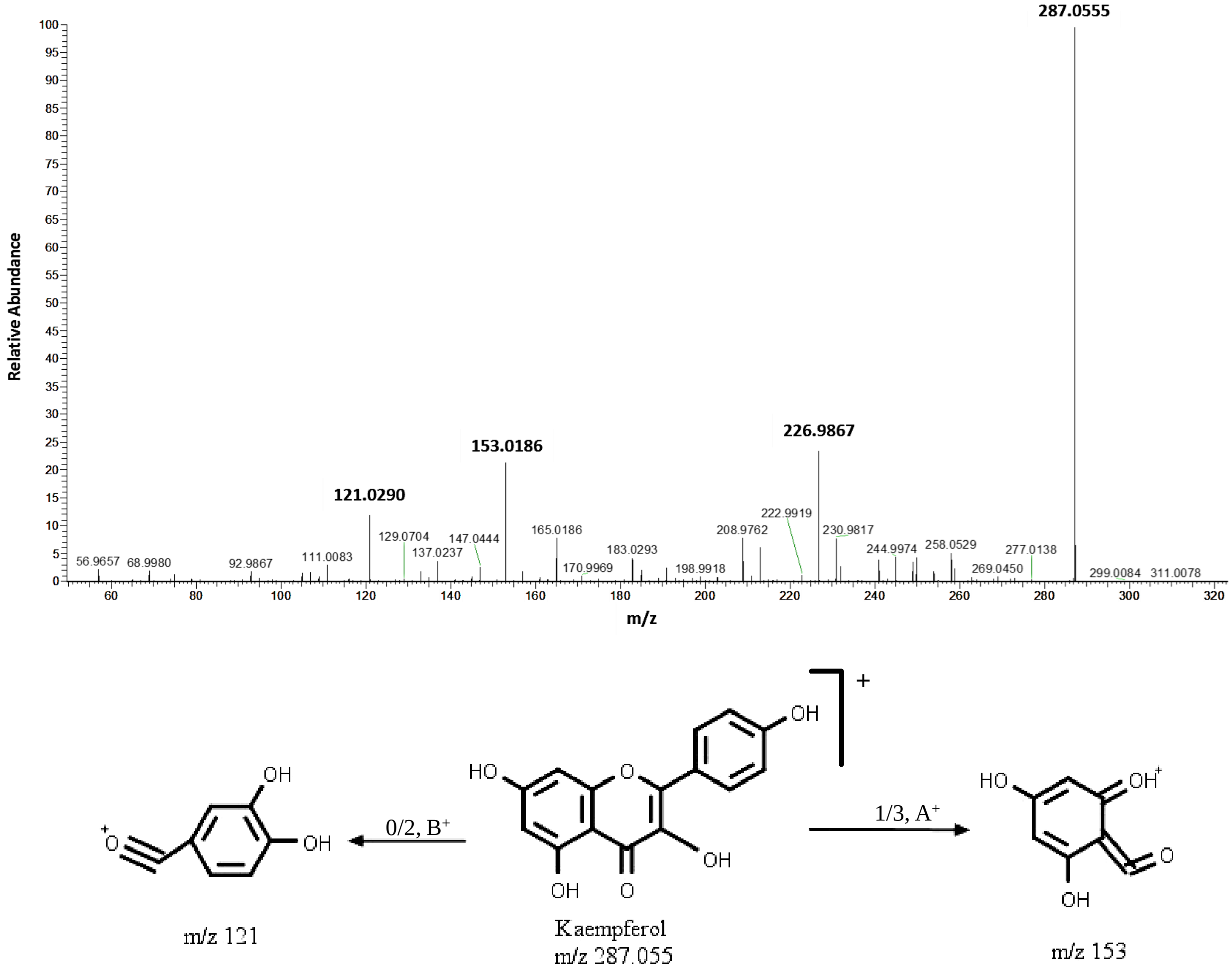

Figure S2: Chemical Structure of Kaempferol and the major possible mass fragmentation

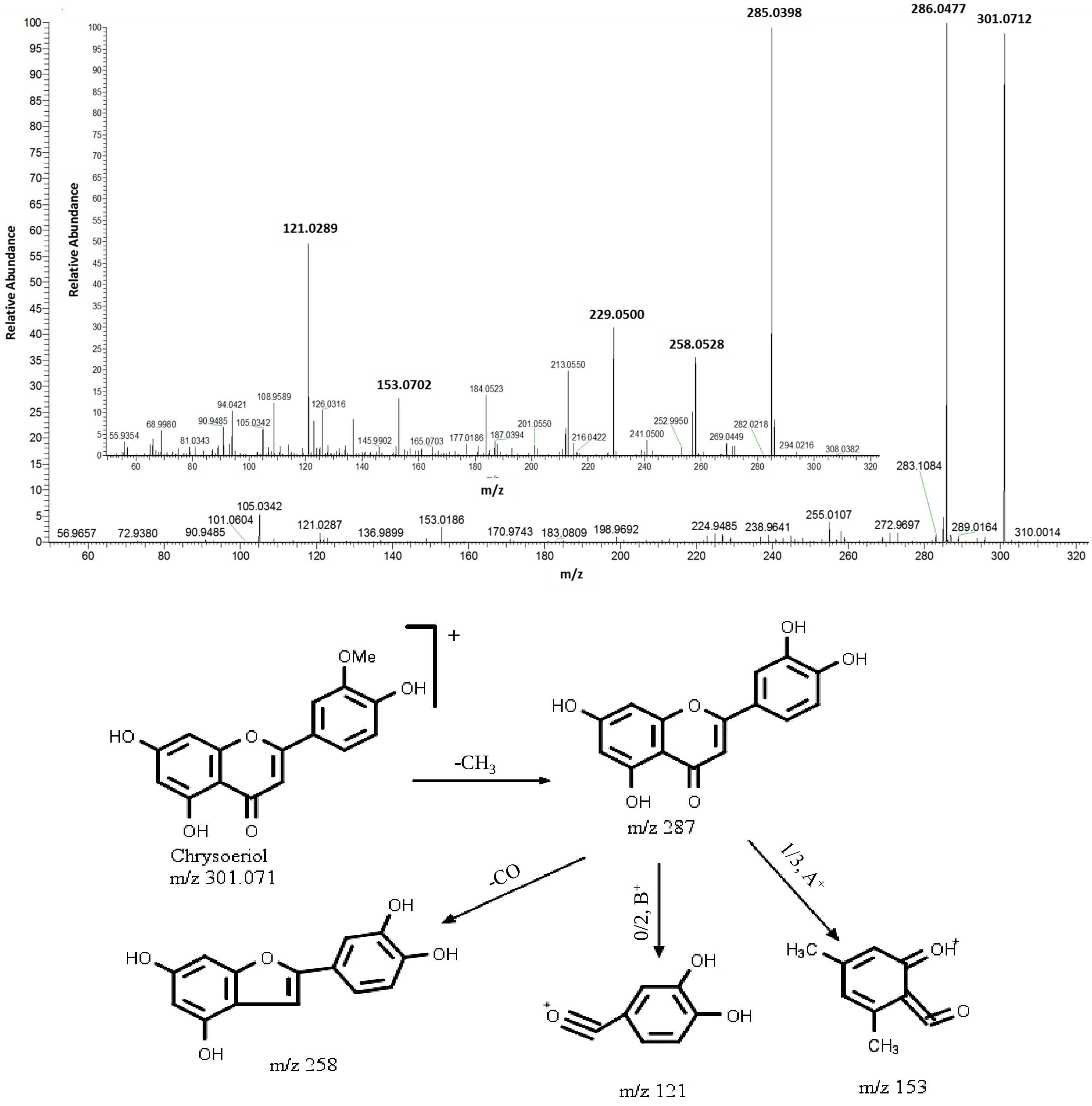

Figure S3: Chemical Structure of chrysoeriol and the major possible mass fragmentation

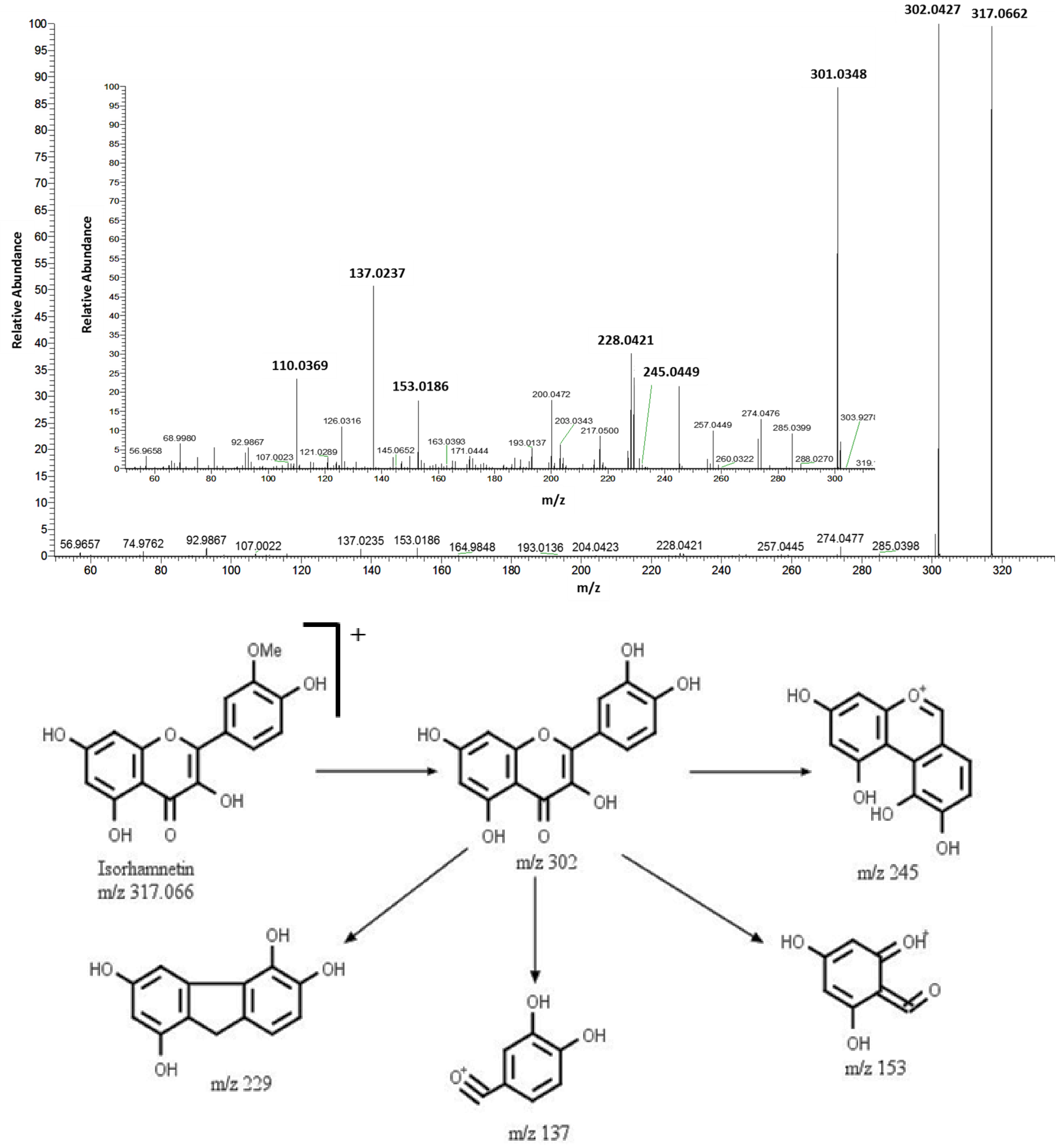

Figure S4: Chemical Structure of isorhamnetin and the major possible mass fragmentation

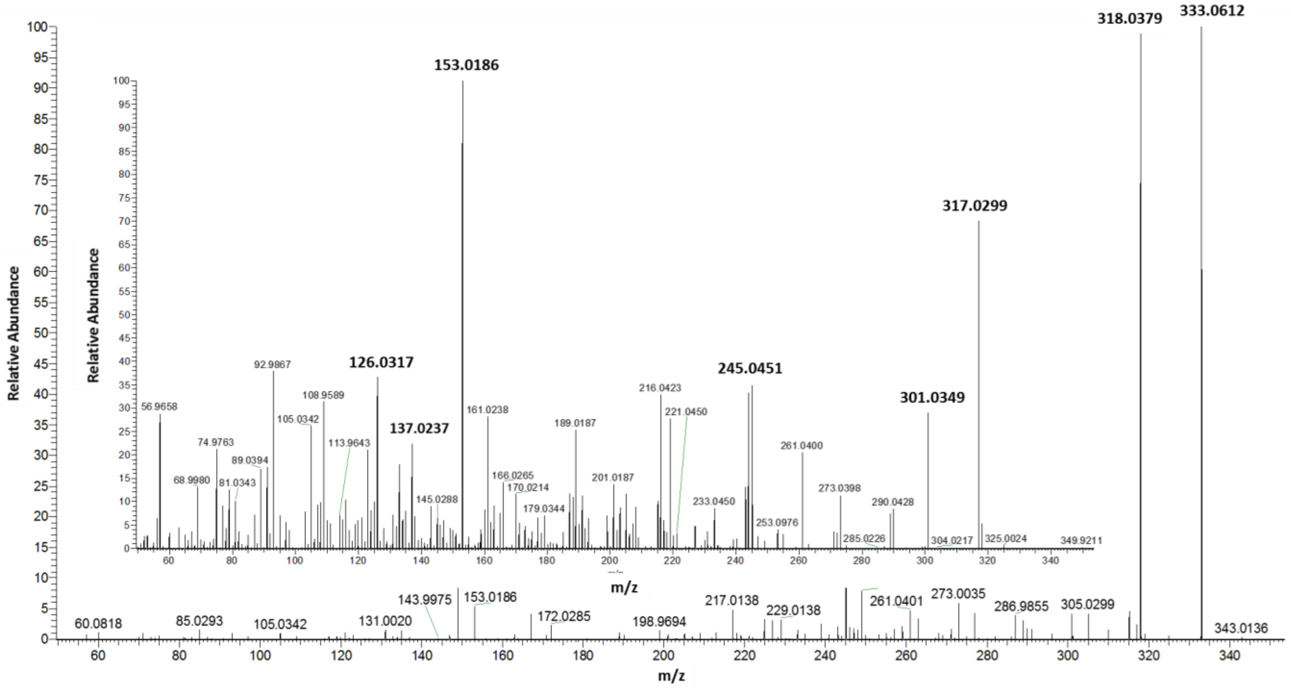
Relative Abundance
m/z
318.0379
333.0612
153.0186
317.0299
126.0317
245.0451
301.0349
137.0237
92.9867
108.9589
56.9658
105.0342
113.9643
74.9763
89.0394
68.9980
81.0343
145.0288
161.0238
166.0265
170.0214
179.0344
189.0187
201.0187
216.0423
221.0450
233.0450
253.0976
261.0400
273.0398
290.0428
285.0226
304.0217
325.0024
349.9211
60.0818
85.0293
105.0342
131.0020
143.9975
153.0186
172.0285
198.9694
217.0138
229.0138
261.0401
273.0035
286.9855
305.0299
343.0136

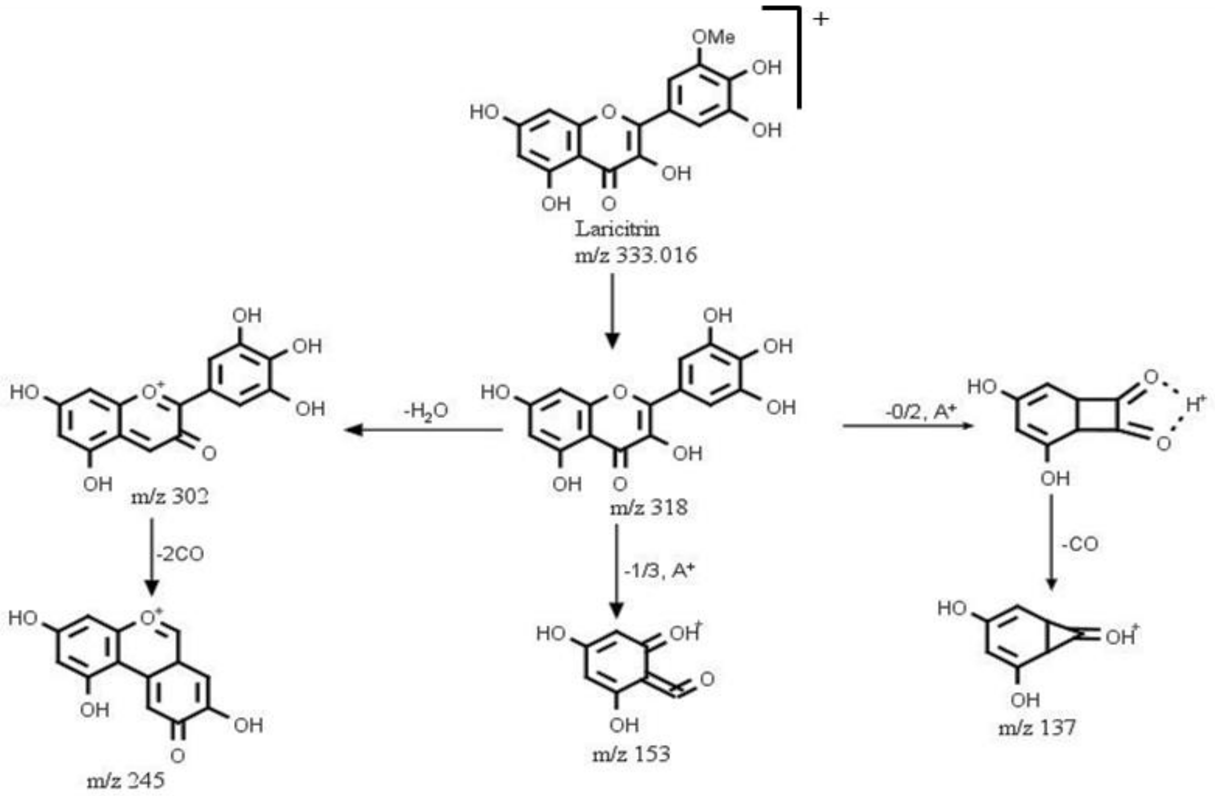
Laricitrin
m/z 333.016
-H2O
-0/2, A+
m/z 302
m/z 318
-2CO
-1/3, A+
-CO
m/z 245
m/z 153
m/z 137

Figure S5: Chemical Structure of laricitrin and the major possible mass fragmentation

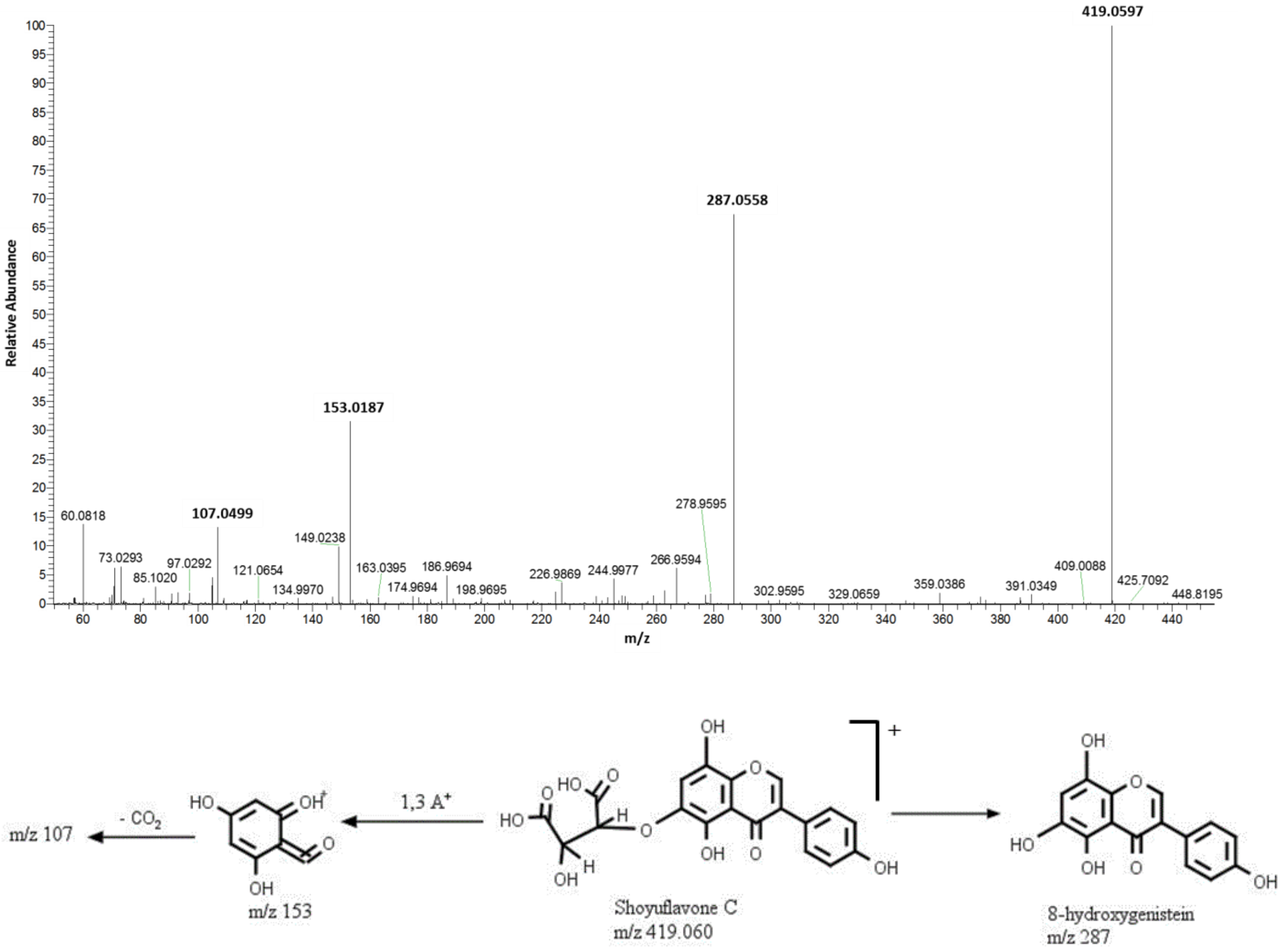

Figure S6: Chemical Structure of shoyuflavone C and the major possible mass fragmentation

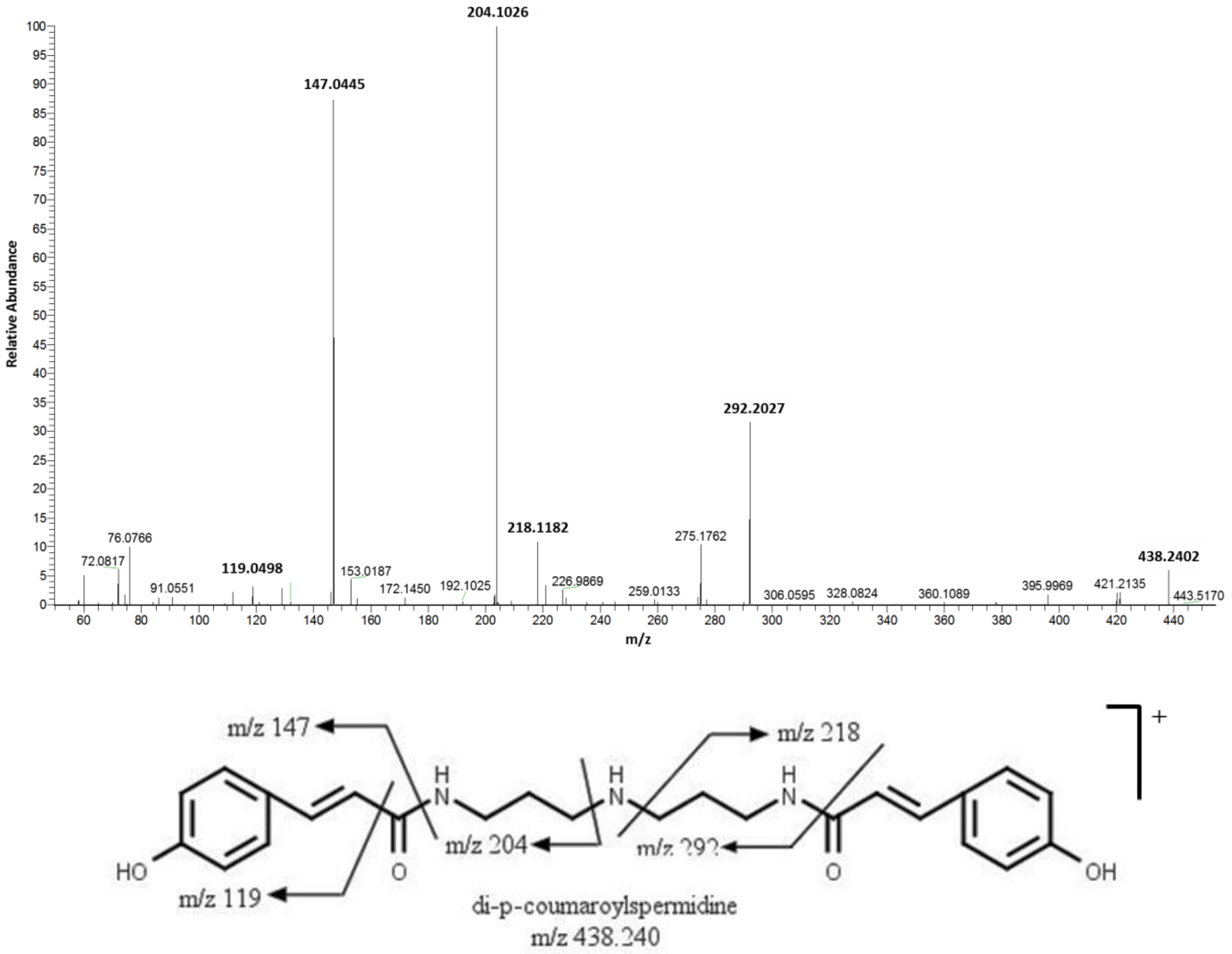

Figure S7: Chemical Structure of di-*p*-coumaroylspermidine and the major possible mass fragmentation

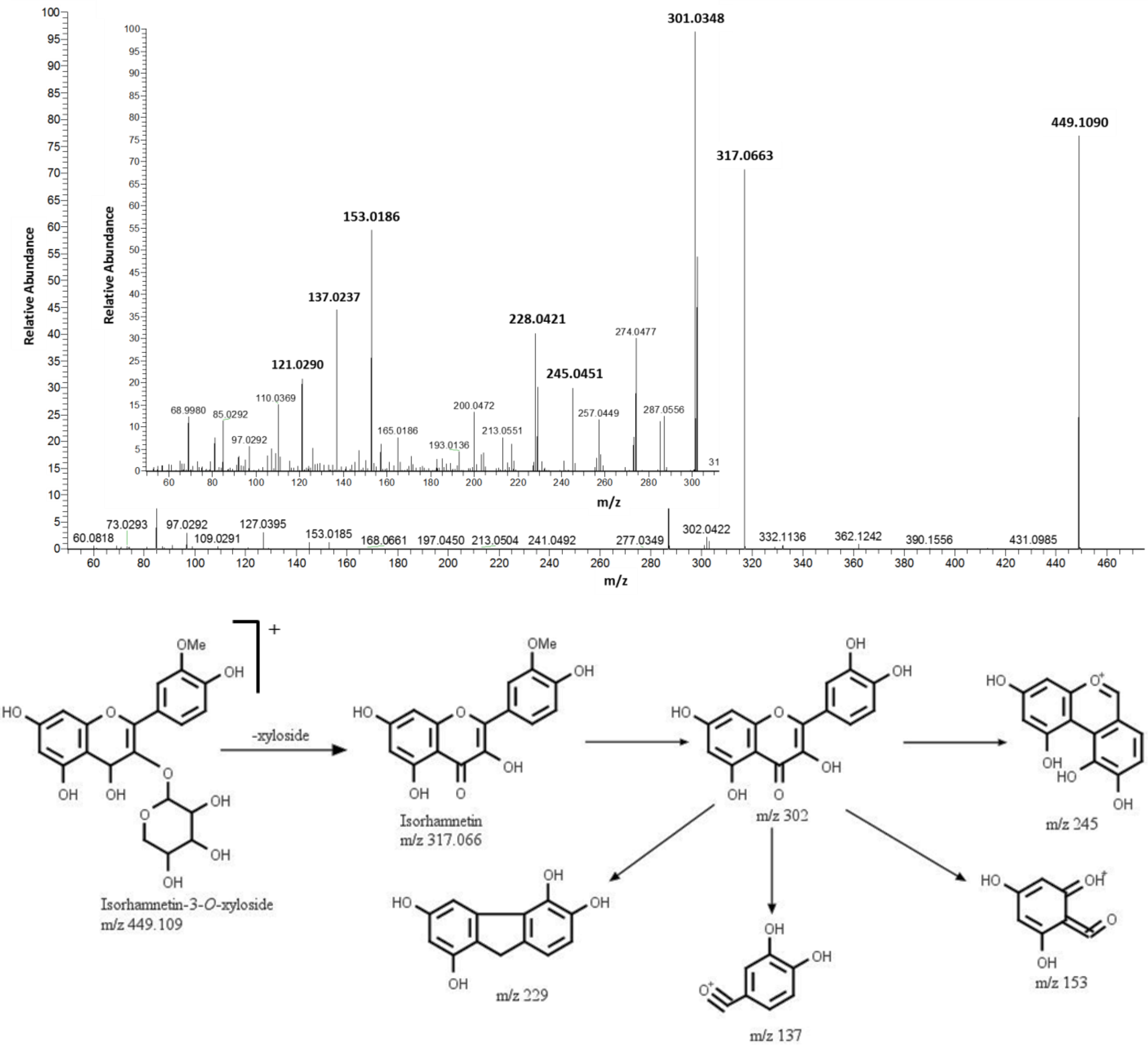

Figure S8: Chemical Structure of isorhamnetin-3-*O*-xyloside and the major possible mass fragmentation

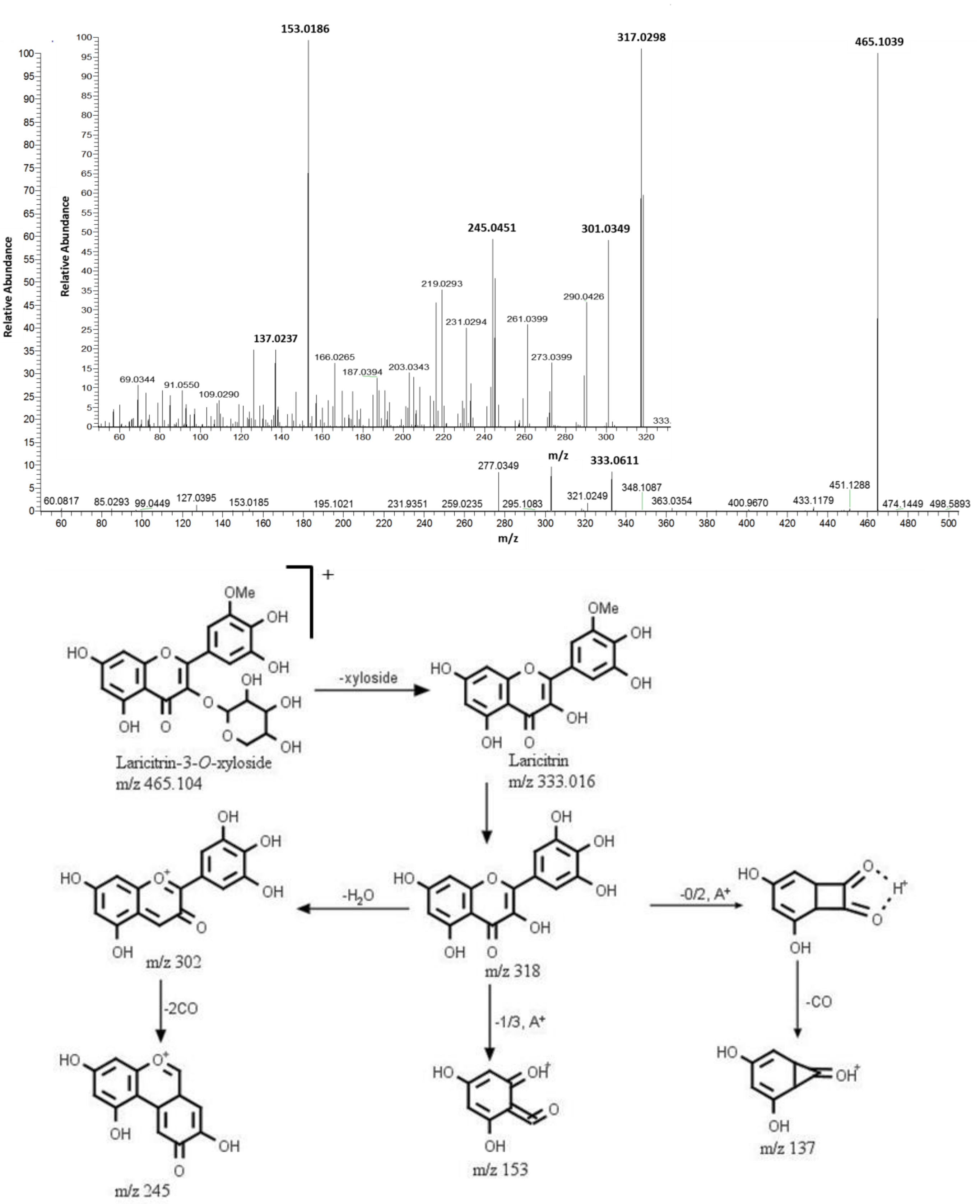

Figure S9: Chemical Structure of laricitrin-3-*O*-xyloside and the major possible mass fragmentation

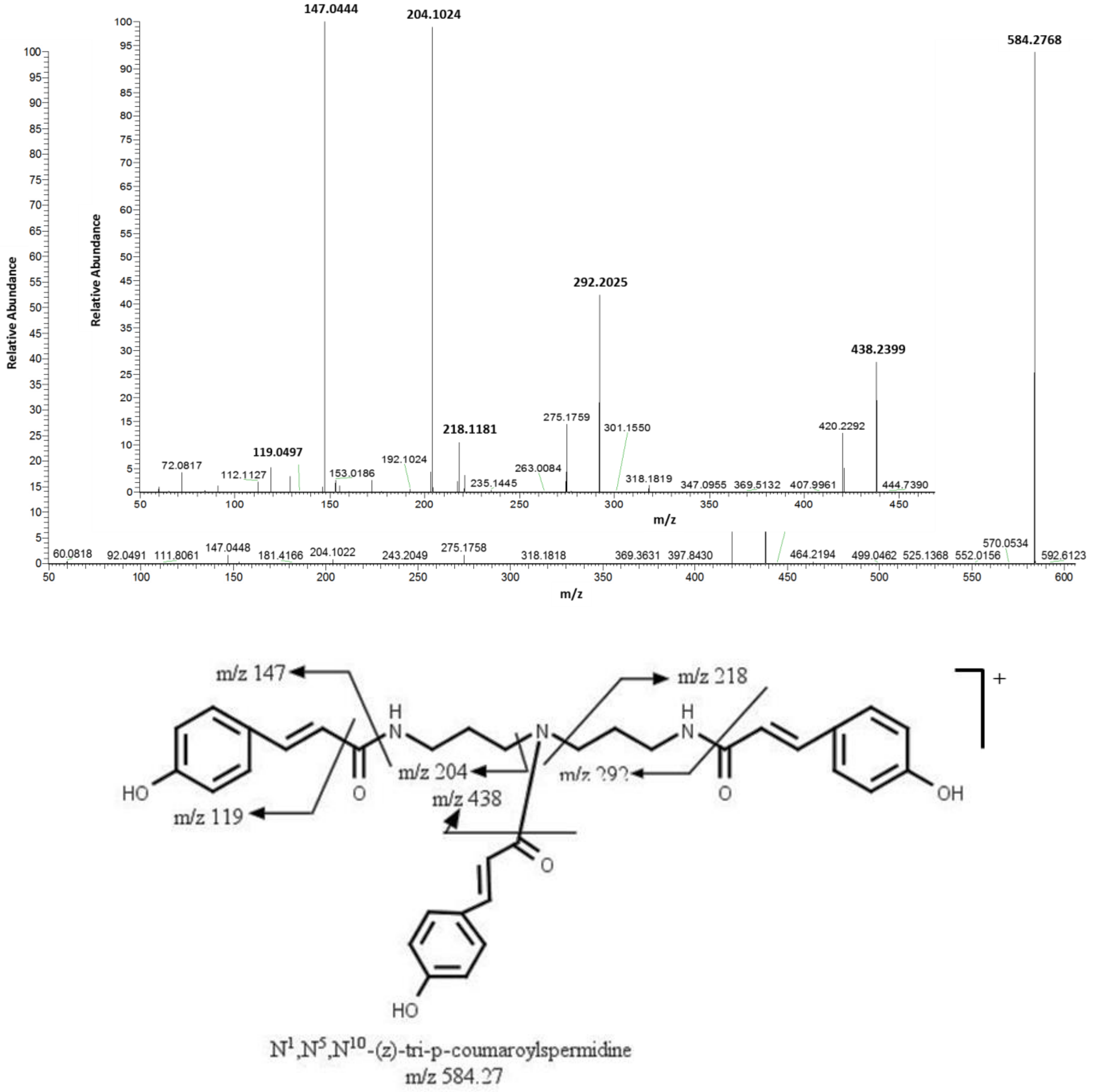

Figure S10: Chemical Structure of N^1,N^5,N^{10}-(z)-tri-*p*-coumaroylspermidine and the major possible mass fragmentation

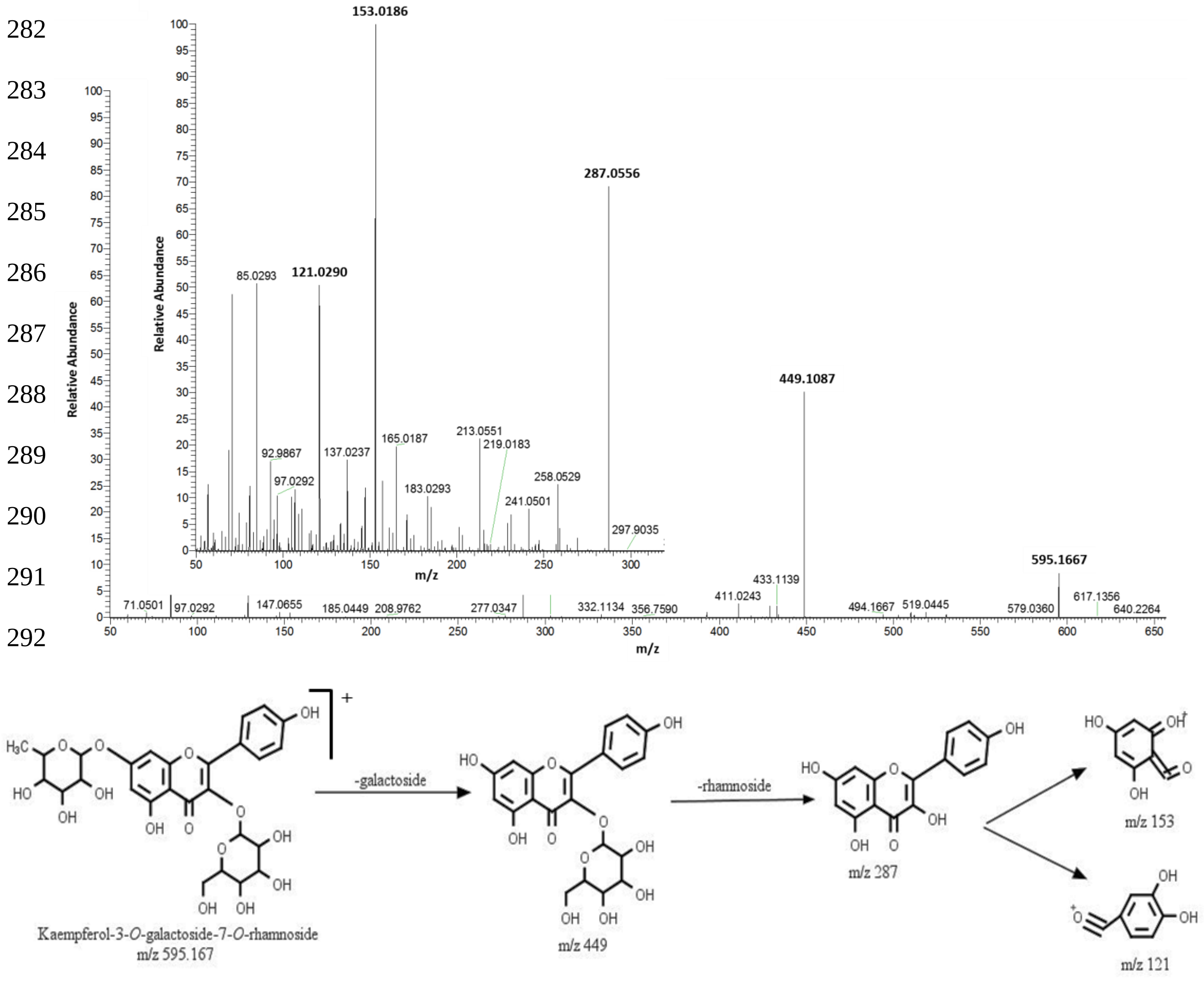

Figure S11: Chemical Structure of kaempferol-3-*O*-glactoside-7-*O*-rhamnoside and the major possible mass fragmentation

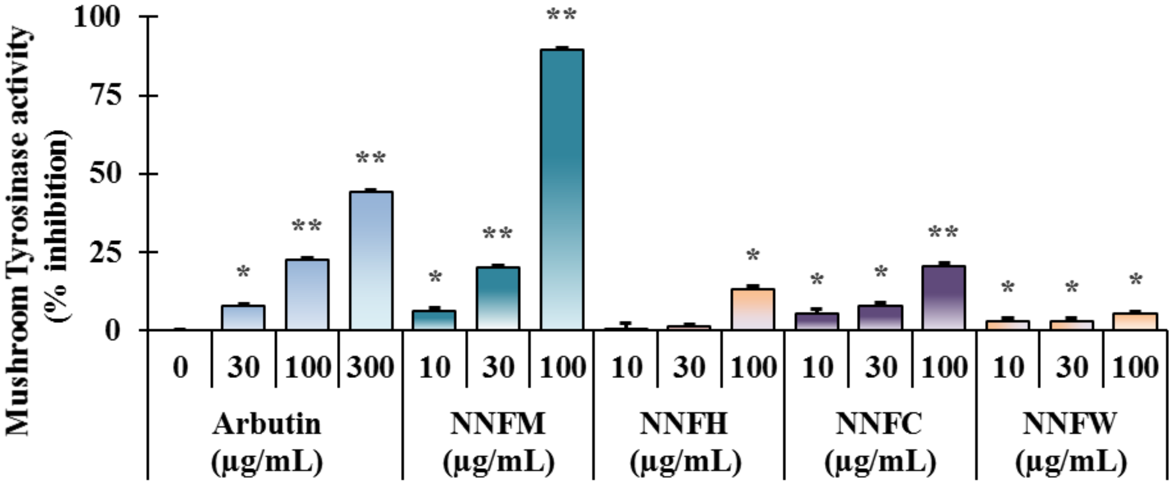

Figure S12: Mushroom tyrosinase inhibition activities of methanolic extract and various organic solution fractions of Nymphaea nouchali flowers. Values are expressed as the mean ± SD (n=3). *p < 0.05 and **p < 0.01, significantly different from control, using student's t-test.

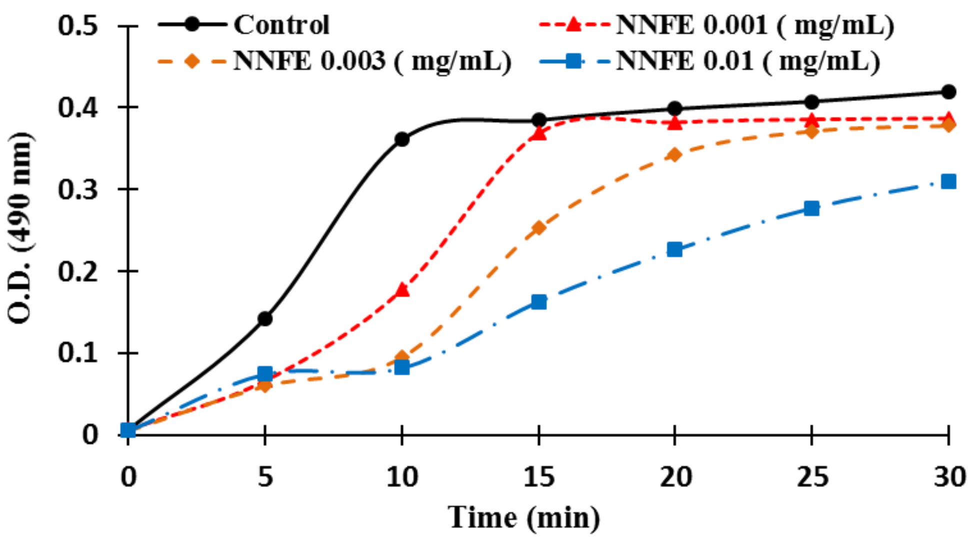

Figure S13: Mushroom tyrosinase inhibition kinetics of ethyl acetate fractions of Nymphaea nouchali flowers (NNFE) in which substrate is L-DOPA. Values are expressed as the mean ± SD (n=3).

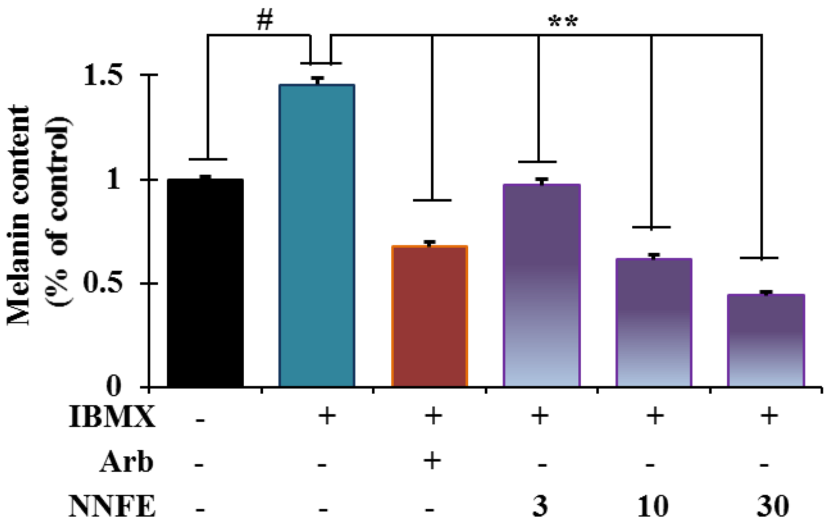

Figure S14: Cells were cultured with NNFE (3-30 μg ml^{-1}) for 3 days. IBMX induced melanin content were measured. Values are expressed as the mean ± SD (n=3). #$p < 0.05$ significantly different from control and **$p < 0.01$, significantly different from IBMX-induced control, using student's t-test.

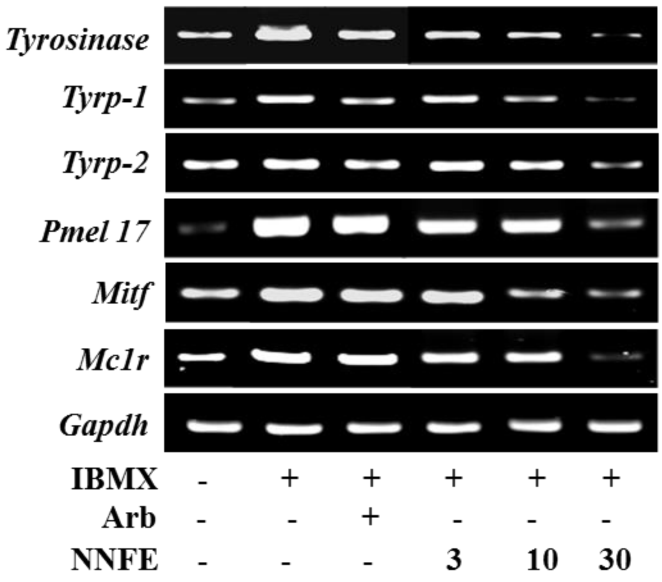

Figure S15: Cells (5×10^5 cells ml^{-1}) were cultured for 24 h, and the medium was then replaced with fresh medium containing the indicated concentrations of NNFE or arbutin for 24 h. mRNA was extracted using TRIzol and mRNA expressions was analyzed by RT PCR.

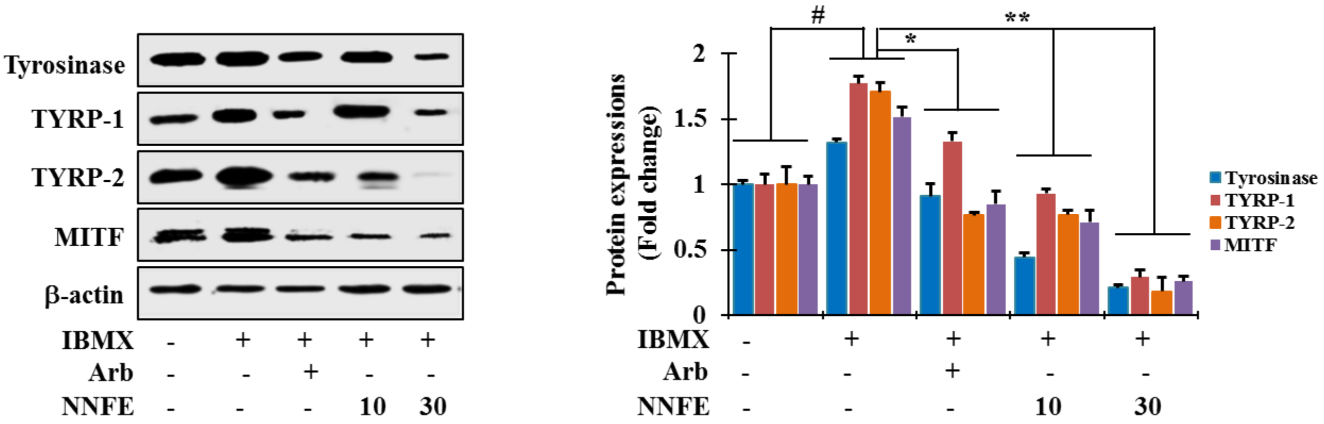

Figure S16: Cells (1×10^5 cells ml^{-1}) were cultured for 24 h; medium was replaced with fresh medium containing the indicated concentrations of NNFE or arbutin for 3 days. Total cell lysates were extracted and assayed by western blotting using antibodies against tyrosinase, TYRP-1, TYRP-2, and MITF Equal protein loadings were confirmed using β-actin. Arb: Arbutin. Statistical analysis of the band intensity of tyrosinase, TYRP-1, TYRP-2, and MITF obtained by western blot analysis. #$p < 0.05$ significantly different from control and **$p < 0.01$, significantly different from IBMX-induced control, using student's t-test.

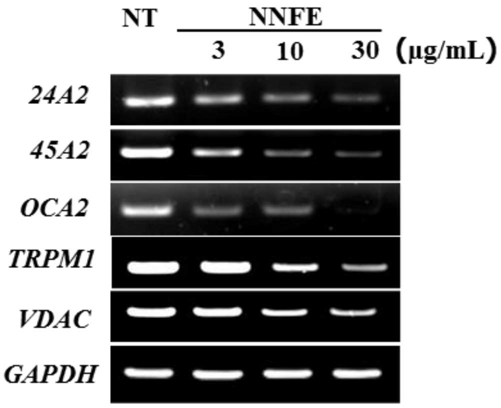

Figure S17: Cells (5×10^5 cells ml^{-1}) were cultured for 24 h, and the medium was then replaced with fresh medium containing the indicated concentrations of NNFE for 24 h. mRNA was extracted using TRIzol and mRNA expressions was analyzed by RT PCR.
